# Shades of smiles: creating variants of smiles from neutral images of real individuals - method and validation

**DOI:** 10.1007/s00426-026-02263-z

**Published:** 2026-03-19

**Authors:** Jin Gao, Werner Sommer, Rasha Abdel Rahman, Wei-Jun Li

**Affiliations:** 1 Department of Psychology, Humboldt- Universität zu Berlin, Berlin, Germany; 2 Institute of Psychological and Brain Sciences, Liaoning Normal University, Dalian, China; 3 Department of Physics and Life Science Imaging Center, Hong Kong Baptist University, Hong Kong, China; 4 Faculty of Education, National University of Malaysia, Kuala Lumpur, Malaysia

## Abstract

Despite their crucial role in emotion research, facial expression databases primarily contain stimuli of basic emotions, rather than subtle, socially nuanced ones. Here, we introduce a rigorous yet easily applicable pipeline for synthesizing fine-grained emotional expressions—specifically, reward, affiliative, and dominance smiles—from neutral photographs, guided by Facial Action Coding System (FACS) principles and the perspective that expressions serve as social signals. From neutral images of 90 individuals (45 female, 45 male), we generated five expressions each (including neutral and disgust), and mirrored each image to address hemiface biases. In an online validation study, 13 participants rated each image on emotional content, arousal, and plausibility, yielding 26 ratings per expression and model. Rating results show that (1) reward smiles were most reliably recognized, while affiliative and dominance smiles tended to be confused with related expressions and that (2) arousal and plausibility ratings varied systematically across expression types and model gender. In summary, the suggested expression generation pipeline and the validated stimuli offer a robust method and a scalable dataset for research on fine-grained emotion recognition and emotional communication.

## Introduction

Facial expressions convey a wide range of affective states, including feelings, intentions, and desires (Horstmann, 2003) and can be precisely described by specific patterns of muscle activity, referred to as Action Units (Ekman & Rosenberg, 1997; Klingner & Guntinas-Lichius, 2023). These properties have enabled the development of standardized databases of stimuli representing facial expression that serve as valuable research tools. Yet, acquiring diverse non-standard expressions remains challenging. In this study, we suggest a solution for obtaining non-standard expression stimuli based on the artificial creation of face stimuli with custom-defined expressions derived from photographs of individuals displaying neutral expressions.

Unless cartoon faces are used, most realistic facial emotion stimuli have been sourced from real human expressors (Ekman, 1976; Olszanowski et al., 2015; Tracy et al., 2009). For a review, see Dawel et al. (2022). However, integrating large datasets with high-quality expression stimuli presents significant challenges. For instance, AffectNet—a database of facial affect compiled from the internet using numerous emotion-related search queries—comprises over one million images with faces and facial landmark points (Mollahosseini et al., 2019). Nonetheless, these images are typically captured under uncontrolled conditions with considerable variability in lighting, pose, resolution, and background, and only a fraction of images depict a given emotion accurately. Specifically, only about 48% of images queried for “happy” expressions were annotated as happiness; percentages for other emotions were even lower (below 20%; see Mollahosseini et al., 2019, Table 4). In contrast, databases like the MPI Facial Expression Database (Kaulard et al., 2012) and the McGill Face Database (Schmidtmann et al., 2020) provide highly controlled stimuli obtained from trained models. However, training these expressors to reliably produce specific Action Units (AUs) or nuanced expressions—such as shame or composite expressions like a sad smile—requires considerable time and effort, and not all posers achieve consistent results (Mehu et al., 2012; Tottenham et al., 2009). For instance, the MPI Facial Expression Database includes an impressive array of 55 natural emotional and conversational expressions elicited through a method-acting protocol, in which participants were provided with specific everyday scenarios designed to evoke natural and authentic expressions; however, it comprises only 19 German models (Kaulard et al., 2012) Similarly, the McGill Face Database features 93 expressions of mental states but is based on merely two professional expressors (Schmidtmann et al., 2020). Consequently, for specialized research or specific applications, even these extensive databases are limited.

Advances in generative adversarial networks (GANs) and related AI-driven methods have substantially improved the generation of high-quality facial stimuli (Kammoun et al., 2022). For instance, Deepfake technologies, based on GAN architectures, can produce highly realistic face images that are often indistinguishable from real photographs (Tolosana et al., 2020). Similarly, GANimation (Pumarola et al., 2020) offers the ability to manipulate facial Action Units (AUs) to generate expressions in a controllable manner. However, while both methods excel when the input closely matches the training data distribution, they tend to falter when applied to faces with distinctive structures or when generating subtle, nuanced emotional expressions. In particular, Deepfake technologies, although highly convincing in overall face appearance, often lack precise control over specific emotional expressions. Likewise, outputs from GANimation can appear distorted or unrecognizable when dealing with complex or unfamiliar facial features. These limitations, rooted in training biases, restrict the applicability of these methods in research contexts that require a large set of subtle, systematically varied emotional expressions across different individuals.

Facial modelling and animation techniques from computer vision and graphics provide another solution for generating extensive sets of emotional facial expressions. Unlike physics-based simulations that rely on complex geometric and muscular representations (Behrouzi et al., 2025; Rai et al., 2024), the FaceGen software suite—particularly FACSGen—offers an accessible and intuitive platform for AU manipulation. FACSGen 2.0 integrates advanced algorithms with a user-friendly interface to map photorealistic textures onto virtual faces, enabling the precise generation of both static and dynamic expressions. Krumhuber et al. (2012) demonstrated the utility of this software in producing AU-defined facial expressions, including canonical emotions (e.g., happiness, anger, fear) and less common social emotions (e.g., embarrassment, contempt), across various genders and ethnicities using grayscale faces.

Building on the work of Krumhuber et al. (2012), the present study focuses on three specific types of smiles—reward, affiliative, and dominance—that commonly function as social signals in interpersonal contexts (Hareli & Hess, 2012). These smiles are defined by distinct Action Unit (AU) configurations (Martin et al., 2017; Niedenthal et al., 2010). Reward smiles are displayed to express happiness, reward others or to communicate positive experiences or intentions; they involve the activation of the Lip Corner Puller (AU12), Inner-Outer Brow Raiser (AU1-2), Sharp Lip Puller (AU13), and Dimpler (AU14) (Rychlowska et al., 2017). Affiliative smiles are shown to signal appeasement and create and maintain social bonds and also involve AU12 but are distinguished from Reward smiles by activating the Lip Pressor (AU24) and Dimpler (AU14). Dominance smiles are displayed to negotiate status within and across social hierarchies; they also feature AU12 but asymmetrically, involving either the right or left Lip Corner Puller (AU12R or AU12L). They are also characterized by the Upper Lid Raiser (AU5), Nose Wrinkler (AU9), Cheek Raiser (AU6), and Upper Lip Raiser (AU10). To allow for manipulation checks and to differentiate positive from at least one negative expression, we also included disgust expressions. Disgust, a negative expression, shares the Nose Wrinkler (AU9) with the dominance smile (Pochedly et al., 2012). Furthermore, neutral expressions were derived from the model faces with the same pipeline but without AU activation, allowing these expressions to serve as experimental baseline.

While Krumhuber et al. (2012) pioneered AU-based facial expression generation using FACSGen 2.0, the present study addresses key limitations in their study and extends it to broader applications. First, whereas Krumhuber et al. (2012) used a small number of expressors (at most four expressors per experimental condition), our stimuli were derived from a much broader range of real front-facing neutral portraits sourced from multiple databases. These images were converted into standardized head expressors while preserving identity-specific features. Although this study focused on a single racial group to ensure experimental control, our method is scalable to more diverse populations. Second, although Krumhuber et al. (2012) included a few less-common social expressions (e.g., embarrassment, contempt), their work did not provide a comprehensive pipeline for generating and validating nuanced, socially meaningful variants of expressions. In contrast, our integrated pipeline systematically produces and validates three subtle smile types—reward, affiliative, and dominance—each capturing distinct social signals such as appeasement versus assertiveness. Third, building on the validation approach used by Krumhuber et al. (2012), we evaluated recognition accuracy, perceived arousal, and plausibility. Crucially, we introduced the arcsine-transformed Unbiased Hit Rate (UHR), a response-bias-resistant metric, and incorporated it into a composite index combining plausibility ratings, allowing for a more comprehensive assessment of model performance. Finally, to support reproducibility, we provide open-source validation code and share all stimuli derived from our in-house images.

Building on these methodological extensions, the present study aimed to establish a systematic pipeline for generating socially nuanced emotional expressions from neutral portraits and to validate a set of synthesized smile stimuli created through this approach. We hypothesised that (1) participants would reliably recognize and accurately identify the distinct emotional expressions depicted in the synthesized facial images, that is, “reward smiles,” “affiliative smiles,” “dominance smiles,” “disgust,” and “neutral expressions.” The accuracy rates for identifying these expressions were expected to be significantly better than chance, indicating clear and distinguishable emotional contents. (2) Smiles and disgust were expected to be associated with higher perceived arousal levels than neutral expressions. (3) The synthesized images were expected to be perceived as highly plausible, with participants judging the images as being derived from photographs of real humans.

## Methods

### Construction stage

**Fig. 1:**
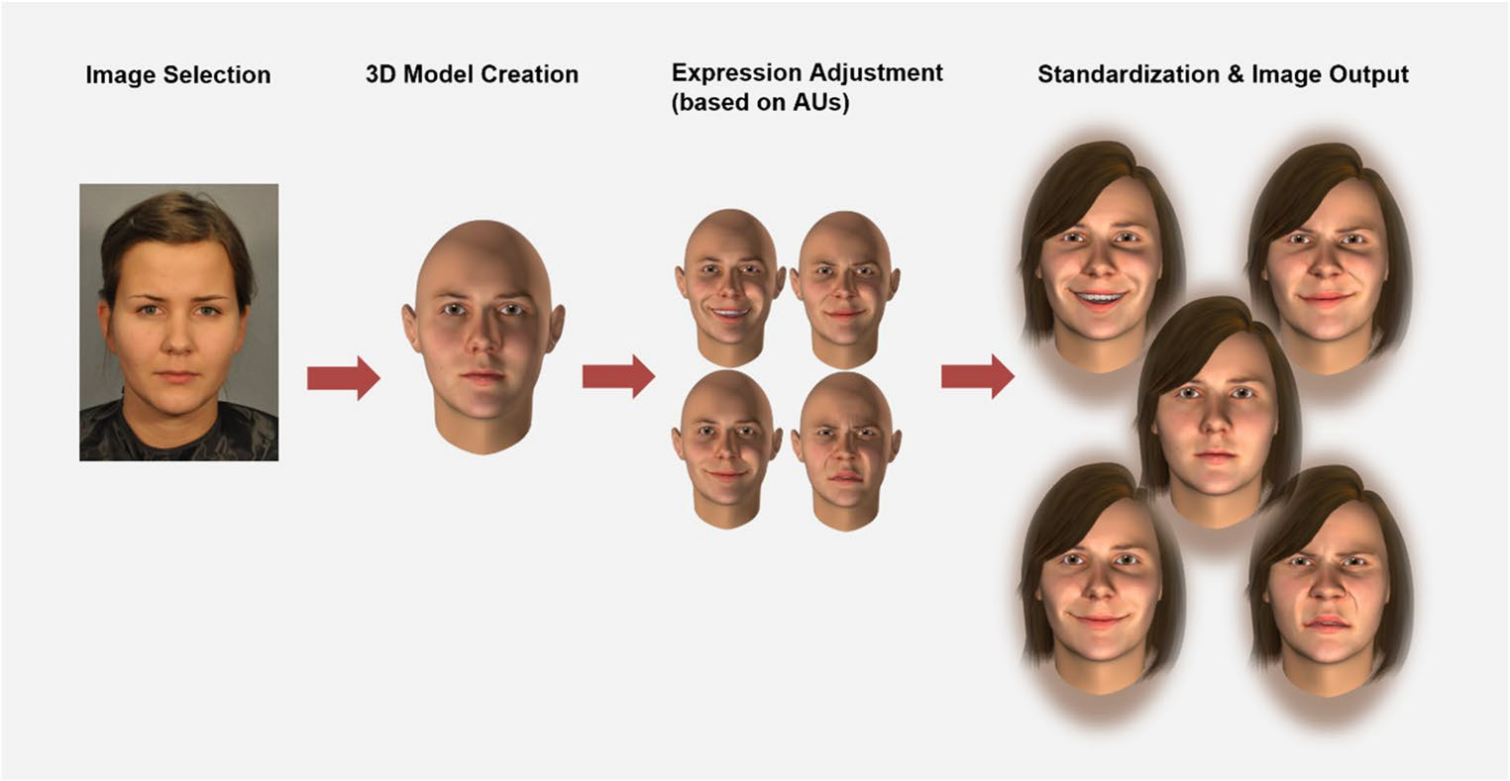
Pipeline for generating facial expression stimuli. *Note.* The final output includes five expressions: neutral (centre), reward smile (top left), affiliative smile (bottom left), dominance smile (top right), and disgust (bottom right)

To create the emotional expression stimuli, we followed a structured series of steps as illustrated in Fig. 1.

#### Step 1: selection and preparation of database sources

As original images we selected 90 high-resolution front-facing photographs of Caucasian individuals with neutral facial expressions from several sources (DeBruine & Jones, 2017; Ebner et al., 2010; Ma et al., 2015; Rellecke et al., 2012). Stimuli comprised 45 male and 45 female expressors, with mean ages of 25.7 and 26.3 years, respectively. While there were slight variations in parameters across picture sources (e.g., the colour of backgrounds and the size of the pictures), they were sufficiently similar to provide a consistent basis for the generation of head models since they were, taken under similar shooting conditions and ensured clear visibility of individual facial features. Besides, we selected only models without beards or non-face features, such as, scars, tattoos or piercings, jewellery, glasses, makeup or any clothing that covered the neck.

#### Step 2: generation of head models

We utilized the FaceGen Modeller, a part of the FaceGen software suite (Roesch et al., 2011), allowing to generate a virtually infinite number of 3D facial identities as head model. The selected photographs were imported into FaceGen Modeller software. The software extracted facial geometry and skin texture information from these images, constructing detailed 3D head models with realistic skin textures and individual-specific features. Specifically, photorealistic skin texture was mapped onto the face. Different texture layers (i.e., diffuse colour, ambient occlusion, and gloss and normal maps) were also included, which are combined during the rendering stage to achieve the final appearance (Krumhuber et al., 2012).

#### Step 3: generating and adjusting facial expressions based on action units (AUs)

FACSGen 2.0 software (Krumhuber et al., 2012) allows detailed adjustment and combining various facial expressions based on Action Units (AUs), enabling researchers to simulate a range of emotional expressions without altering the identity of the expressors. We began the process by selecting specific combinations of AUs that correspond to the targeted facial expressions “reward smile,” “affiliative smile,” “dominance smile,” and “disgust.” The AU combinations for each expression (see Table 1) were selected based on the smiling prototypes described by Martin et al. (2017) and the disgust prototype (Pochedly et al., 2012). Additionally, since all original portraits displayed neutral expressions, the ‘neutral expression’ of the 3D models was directly generated from the imported photographs without any manipulation of AUs.

**Table 1:** Targeted facial expressions for each emotion and their corresponding action units (AUs)Targeted facial expressions for each emotion and their corresponding action units (AUs)

Expression	AU combinations
reward smile	**AU1 + AU2 +** AU6 + AU7 + **AU12 + AU13 + AU14 +** AU20 + AU26 + ahh + i + k
affiliative smile	AU6 + AU7 + **AU12 +** AU13 + **AU14 +** AU20 + **AU24**
dominance smile	AU2 + AU4 + (AU1 + 2+4) + **AU5 + AU6 +** AU7 + **AU9 + AU10 + AU12+** AU14_Unilateral + AU25 + AU17
disgust	AU4 + (AU1 + 4) + **AU6 +** AU7 + AU9 + **AU10 +** AU11 + AU16 + AU15 + AU17 + AU20 + AU24

*Notes.* AUs in boldface are considered essential for inclusion in the final portrayal of a given expression; AUs in normal typeface can occur at any level of intensity. AUs named “ahh”, “I”, “k”, and those in parentheses are pre-defined combinations of AUs in FACSGen 2.0, which can be used to fine-tune the expressions to better approximate their prototypes.

In FACSGen 2.0, each AU is represented by a slider controlling the magnitude of the morph target, which gradually transforms the original neutral expression (0%) into the fully activated target expression (100%). We used the slider-based controls to adjust the intensity of the AUs specified for the target emotion, fine-tuning them to closely match the prototype of each specific expression. This was necessary because setting the AU activity at a preset level for all faces may induce artificial looking results. In adjusting the AU strengths we followed the principle of keeping the expressions as natural as possible without unnatural facial distortions, such as teeth-lip intersections or excessive muscle activation. For AU12—activated in all three types of smiles and playing a crucial role in defining the characteristic look of each smile—the intensity was uniformly set to “80%” for all three smiles (without manual fine-tuning). It is important to note that FACSGen does not provide asymmetric AU12 activation, which is considered to be crucial for the dominance smile (Martin et al., 2017; Niedenthal et al., 2010). To achieve this effect, we employed a compensatory method by combining “dragging one side of the mouth corner upward in FaceGen Modeller on the face picture” and increasing the intensity of AU14_Unilateral in FACSGen to approximate the asymmetric AU12 effect.

#### Step 4: standardization of display parameters and generation of static images

The original hairstyle and hair colour of the individuals in the photographs cannot be retained during the 3D model generation process because the FACSGen 2.0 software does not support importing or replicating external features such as hairstyle and hair colour. To further standardize the images, we ensured uniformity of hairstyle and hair colour of the generated head models. Note, there are many hair styles and colours available in FACSGen that could be used. For present purposes, all individuals of a given sex were assigned the same sex-typical hairstyle and all models, regardless of sex, were given a uniformly brunette hair colour. Additionally, given evidence fornegative frequency-dependent sexual selection on eye colour in Europeans (Uhl & Carter, 2023),the stimulus set was designed to reflect broad phenotypic variation (40% brown, 30% blue, 20%green, 8% grey) rather than a single dominant trait. Display parameters, including frontal face orientation, taupe background, and lighting conditions, were uniformly set for all 3D models. To simplify evaluation and communication, we exported 2D front view projections of the models at a resolution of 800 × 1200 pixels with 32-bit depth. The 2D projections captured the key features of the 3D models in a single, clear image, making it straightforward to identify essential characteristics and potential issues. These images were then used for validation.

## Validation stage

### Participants

As detailed in our pre-registration^1^, we conducted an a priori power analysis in G*Power 3.1 for our mixed‐design ANOVA (within‐between interaction) with the following parameters: medium effect size (*f* = 0.25), *α* = 0.01, power (1 − *β*) = 0.90, total groups = 10 (5 lists × 2 versions), repeated measures = 5 (emotion categories), assumed correlation among repeated measures = 0.50, and non-sphericity correction (*ε*) = 1. This yielded a minimum of 80 participants (8 per group). To enhance the precision of item‐level variance and confidence intervals, we increased to *N* = 130 raters (13 per group), exceeding the a priori requirement and ensuring robust power for all planned contrasts.

Accordingly, via Prolific (https://www.prolific.com/), we initially recruited *N* = 135 participants (paid £7.62/hr). All were right-handed adults (18–34 years) with normal or corrected vision, no reported physical or mental health issues, and self‐identified as White to preclude other‐race effects given our Caucasian models (Crookes et al., 2015; Zhao et al., 2024). The study adhered to the Declaration of Helsinki and received ethics approval from the Department of Psychology at Humboldt-Universität zu Berlin. Participants gave informed consent, including agreement to non-dissemination of images. After excluding one rater who reported “Other” gender and four with below‐chance accuracy, the final sample comprised 130 participants: 70 women (*M* = 25.73 years, *SD* = 4.30) and 60 men (*M* = 25.68 years, *SD* = 5.02).

### Experimental stimuli

The images used had been synthesized from portraits of 90 expressors (45 males and 45 females) with neutral expressions (according to the normative data of the sources). For each portrait, five images with different expressions were created: “reward smile,” “affiliative smile,” “dominance smile,” “disgust,” and “neutral expression.” For clarity, the synthesized emotional expressions are described as if they had been displayed by the expressors themselves, although they were generated from their neutral portraits. To control for potential hemiface effects—i.e., asymmetry biases in emotion recognition where observers preferentially attend to the left hemiface (Borod et al., 1997; Busin et al., 2018)—two mirrored versions of each facial image were created, labelled as L-version and R-version, respectively.

o minimize rater fatigue while ensuring comprehensive coverage, the 90 expressors were divided into five groups of 18 (9 males and 9 females each) and assigned to different groups of raters. A Latin Square design (see Table 2) was used to assign each expressor in a group to display only one of the five expressions (reward, affiliative, dominance, disgust, neutral) in either the original or mirrored version, resulting in five different image lists. For each of these five lists, a corresponding mirrored version was created, yielding a total of ten image lists. For example, in List 1, expressors in Groups 1–5 displayed reward, neutral, disgust, dominance, and affiliative expressions, respectively; in List 2, the same expressors displayed neutral, disgust, dominance, affiliative, and reward expressions, respectively. This rotation continued across all ten lists, ensuring that within a given list each expressor appeared with only one expression, but across lists each expressor appeared with all expressions in both L- and R-versions. Each list was rated by a distinct group of 13 participants; hence, every image (i.e. combination of expressor and expression) received 13 independent ratings while preventing any single rater from seeing multiple expressions of the same expressor.

**Table 2:** Latin square design for stimulus assignment

Participant Group	Stimulus Assignment				
	Group 1 (9 M, 9 F)	Group 2 (9 M, 9 F)	Group 3 (9 M, 9 F)	Group 4 (9 M, 9 F)	Group 5 (9 M, 9 F)
List 1 (L-ver) → Group 1 (*n* = 13)	REW	NEU	DIS	DOM	AFF
List 2 (L-ver) → Group 2 (*n* = 13)	NEU	DIS	DOM	AFF	REW
List 3 (L-ver) → Group 3 (*n* = 13)	DIS	DOM	AFF	REW	NEU
List 4 (L-ver) → Group 4 (*n* = 13)	DOM	AFF	REW	NEU	DIS
List 5 (L-ver) → Group 5 (*n* = 13)	AFF	REW	NEU	DIS	DOM
List 6 (R-ver) → Group 6 (*n* = 13)	REW	NEU	DIS	DOM	AFF
List 7 (R-ver) → Group 7 (*n* = 13)	NEU	DIS	DOM	AFF	REW
List 8 (R-ver) → Group 8 (*n* = 13)	DIS	DOM	AFF	REW	NEU
List 9 (R-ver) → Group 9 (*n* = 13)	DOM	AFF	REW	NEU	DIS
List 10 (R-ver) → Group 10 (*n* = 13)	AFF	REW	NEU	DIS	DOM

Note. Each of the 10 participant groups (*n* = 13 per group) viewed one stimulus list containing 90 images (18 expressors × 5 expressions). *L-ver* original version, *R-ver* horizontally mirrored version, *M* male, *F* female. Abbreviations: *REW* reward smile, *AFF* affiliative smile, *DOM* dominance smile, *DIS* disgust, *NEU* neutral expression

### Procedure

The ratings were conducted online using the SoSci Survey platform. Raters were informed that they were taking part in a validation study of emotional pictures of human faces. Before the main task, participants were instructed to adjust their viewing distance to their screens at home to standardize the visual angle of the facial images; the horizontal width of the face image should roughly correspond to the combined width across index, middle, and ring fingers with arms outstretched. This arrangement yields a visual angle of approximately 4° horizontally, as used in many experiments.

The instructions emphasized that there are no ‘right’ or ‘wrong’ answers and participants were encouraged to base their responses on their spontaneous and subjective opinions but to pay close attention to the (sometimes) subtle differences between the facial expressions. During the practice phase, participants were familiarized with the six expression categories used in the task: reward smile, affiliative smile, dominance smile, neutral, disgust, and other. These categories were operationally defined to support consistent judgment, based on brief descriptions and everyday scenarios (e.g., reward smile: “expressing positive internal happiness, for example, after receiving good news”; affiliative smile: “inviting and maintaining mutually positive and beneficial social bonds, typically expressed when greeting a stranger”; dominance smile: “instantiating, stabilizing, or maintaining dominance in a social relation, for example, when disapproving the ideas or actions of a person viewed as intellectually or physically inferior”; disgust: “rejection or revulsion of something or someone potentially contagious, offensive, distasteful, or unpleasant, for example, seeing someone vomiting”; neutral expression: “a non-expressive posture of the face”; other expression: “not belonging to one of the five previous categories”).

**Fig. 2:**
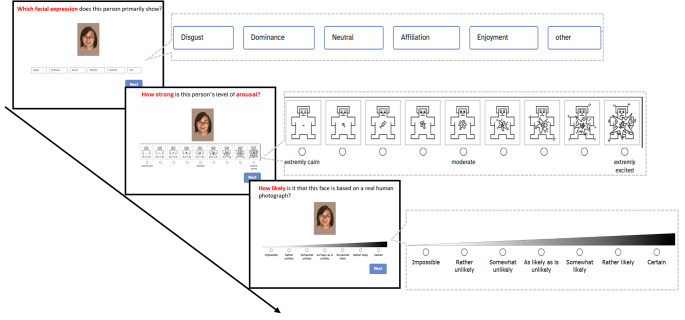
Example of the evaluation process for a single image. *Note.* Participants rated each image on three dimensions presented sequentially: (1) emotional content, (2) arousal, and (3) plausibility. Each question was displayed below the image

As illustrated in Fig. 2, for each image, three questions were posed in order: (1) “Which facial expression does this person primarily show?” (emotional content): Here, the participants should choose one of six options: reward smile, affiliative smile, dominance smile, neutral, disgust, and other. The ordering of the expression labels was kept constant within each rater but was counterbalanced across participants. (2) “How strong is this person’s level of arousal?“. For these arousal ratings we used the Self-Assessment Manikin (SAM; P. Lang, 1980), a pictorial technique with graphic figures representing different levels of arousal, ranging from a completely relaxed, unstimulated, and calm state (depicted by a manikin with eyes closed) to a highly awake, stimulated, and excited state. (3) “How likely is it that this face is based on a real human photograph?” (plausibility). Raters selected one of seven probability levels, ranging from “Impossible” to “Certain”. After each answer, participants clicked the “Next” button to proceed to the subsequent question. For Questions 2 and 3, participants were encouraged to utilize the full range of the provided rating scales.

After completing several practice tasks, participants proceeded to the main experiment. Each image was presented along with the rating questions on the same screen, allowing participants to respond immediately after viewing the image. There was no time limit for completing the ratings, and participants were encouraged to take breaks whenever needed at their own discretion.

Throughout the experiment, five attention check questions were randomly interspersed, as suggested by Prolific, to ensure participants remained engaged and responded attentively. These questions explicitly requested to complete a task in a specific way, for example “The colour test you are about to take part is very simple, when asked for your favourite colour you must select ‘Red’.

## Data analyses and results

In the following we report results for the emotional category ratings as simple hit rates (SHR), unbiased hit rates (UHR), and as confusion matrix. Then we report the results for the arousal and plausibility ratings, followed by an analysis of interrater reliability. All analyses were conducted using R Statistical Software (v4.3.3; R Core Team, 2024).

### Simple hit rate

SHR is a classic accuracy index, which is simple and intuitive (Bänziger et al., 2012). In the current study, SHR were mean percentages of correct responses indicating how often the chosen emotion agrees with the experimenter-intended emotional expression. SHR was calculated separately for each emotion category across all participants and expressors.

Descriptive statistics for SHR across expression categories are summarized in Table 3. The SHR was highest for neutral (*M =* 92.6%, *SD =* 11.3), reward (*M =* 92.1%, *SD =* 14.7), and disgust expressions (*M =* 91.5%, *SD =* 13.5), and substantially lower for affiliative (*M =* 69.2%, *SD =* 29.5) and dominance smiles (*M =* 66.0%, *SD =* 28.8). Given the six-alternative forced-choice format, the theoretical chance level was 16.7%. All mean SHR values were markedly above chance, suggesting that participants were able to recognize the expressions at rates well beyond guessing.

**Table 3:** Mean (M) and Standard Deviation (SD) of recognition accuracy measures by expression

Expression Type	SHR M (%)	SHR SD	UHR M	UHR SD	Chance UHR M	Chance UHR SD
neutral	92.6	11.300	0.880	0.160	0.044	0.012
affiliative	69.2	29.500	0.594	0.305	0.038	0.016
disgust	91.5	13.500	0.892	0.166	0.042	0.009
dominance	66.0	28.800	0.648	0.309	0.031	0.014
reward	92.1	14.700	0.798	0.219	0.049	0.016

*Note*. *SHR* Simple Hit Rate (percentage of correct responses), *UHR* Unbiased Hit Rate, Chance UHR is computed based on the marginal frequencies of the confusion matrix for each expression type (Wagner, 1993).

SHR data were analysed using a binomial (logit) generalised linear mixed model (GLMM). This model was chosen because SHR values are based on trial-level binary outcomes (correct/incorrect), which naturally follow a binomial distribution. Attempts to fit a linear mixed-effects model (LMM)—even after arcsine transformation—violated assumptions of normality and homoscedasticity. A GLMM thus provided a more appropriate framework for analysing the binary nature of the data while accounting for random variability at the rater level. In this model, expression category, group, rater gender, model gender, and version were specified as fixed effects, and rater (case) was included as a random intercept.

The model showed good overall fit (AIC = 8854.1, BIC = 8986.7). Relative to neutral expressions, both affiliative (*β =* − 1.95, *SE =* 0.10, *z =* − 20.27, *p* < 0.001) and dominance smiles (*β =* − 2.12, *SE =* 0.10, *z =* − 22.16, *p* < 0.001) were associated with significantly lower probabilities of a correct response. Disgust (*β =* − 0.16, *SE =* 0.11, *z =* − 1.41, *p =* 0.16) and reward smiles (*β =* − 0.07, *SE =* 0.11, *z =* − 0.58, *p =* 0.56) did not differ significantly from neutral. No significant effects were found for group, rater gender, face gender, or version (all *p* > 0.10). Model diagnostics using the DHARMa package indicated no signs of overdispersion (*p =* 0.984) or zero-inflation (*p =* 0.938), supporting the adequacy of model fit. Tukey-adjusted post-hoc comparisons showed that SHR for affiliative and dominance smiles were significantly lower than for neutral, reward, and disgust expressions (all *p* < 0.001). Additionally, affiliative expressions yielded significantly higher SHR than dominance expressions (*p =* 0.075, marginal).

### Unbiased hit rate

Accuracy estimates are affected by two kinds of biases - the relative number of items for each emotion category presented (presentation bias) and the relative utilization of different emotion categories by the participants (response bias) (Bänziger et al., 2012). UHR is an accuracy index, which considers both biases by calculating “the joint probability that a stimulus is correctly identified (given that it is presented) and that a response is correctly used (given that it is used)” (Wagner, 1993). Here we calculated the UHR following Bijsterbosch et al. (2021) in two steps: First, a choice matrix was created with chosen emotion expressions as columns and intended emotional expressions as rows. Second, the number of ratings in each cell was squared and divided by the product of the marginal values of the corresponding row and column (Note: there were no missing data). Since UHR is a proportion, the values were arcsine-transformed to correct for skewed variances of decimal fractions obtained from count (Bishara & Hittner, 2012; Goeleven et al., 2008). The transformed scores can range from 0 to 1.57, the arcsine of 1, representing perfect identification.

Descriptive statistics for arcsine-transformed UHRs followed a similar pattern with SHRs (summarized in Table 3). Neutral, reward, and disgust expressions achieved high UHRs (e.g., arcsine *M =* 1.42, 1.37, and 1.39 respectively), whereas affiliative and dominance yielded lower performance (arcsine *M =* 1.00 and 1.03). Similarly, unbiased hit rates (UHR) exceeded chance levels across all expressions, further confirming the recognizability of the synthesized emotional stimuli.

To compare the UHRs across emotion categories, we applied a linear mixed effects model (LMM) on arcsine-transformed unbiased UHRs, treating expression category, group, rater gender, model gender, and mirror version as fixed effects, and including a random intercept for each rater (CASE). The model converged with a REML criterion of 732.7. Relative to the reference category (neutral), expression type showed significant effects. Specifically, compared to neutral, the affiliative condition yielded a significantly lower UHR (*β* = −0.425, *SE* = 0.026, *t* = −16.50, *p* < 0.001), as did the dominance condition (*β* = −0.394, *SE* = 0.026, *t* = −15.26, *p* < 0.001). In addition, reward exhibited a small yet significant effect (*β* = −0.052, *SE* = 0.026, *t* = −2.02, *p* = 0.043), whereas disgust did not differ from neutral (*β* = 0.012, *SE* = 0.026, *t* = 0.48, *p* = 0.630). Other factors (face gender, rater gender, version, and group) did not significantly affect UHR (all *p* > 0.10).

Post-hoc pairwise comparisons (Tukey-adjusted) revealed that neutral differed significantly from both affiliative (*estimate* = 0.425, *t* = 16.50, *p* < 0.0001) and dominance (*estimate* = 0.394, *t* = 15.26, *p* < 0.0001), whereas differences between neutral and either reward (*estimate* = 0.052, *t* = 2.02, *p* = 0.2553) or disgust (*estimate* = −0.012, *t* = −0.48, *p* = 0.9890) were not significant. Additionally, affiliative differed significantly from both disgust (*estimate* = −0.437, *t* = −16.98, *p* < 0.0001) and reward (*estimate* = −0.373, *t* = −14.47, *p* < 0.0001), while the difference between affiliative and dominance was non-significant (*p* = 0.7413). Further comparisons (e.g., disgust vs. dominance: *estimate* = 0.406, *t* = 15.74, *p* < 0.0001; dominance vs. reward: *estimate* = −0.341, *t* = −13.24, *p* < 0.0001) underscore the robust impact of expression type on UHR.

Overall, our findings on UHR aligns with SHR: neutral, disgust, and reward conditions yield higher recognition performance than affiliative and dominance, while group assignment, rater gender, model gender, and version have no systematic impact.

### Confusion matrix

**Fig. 3:**
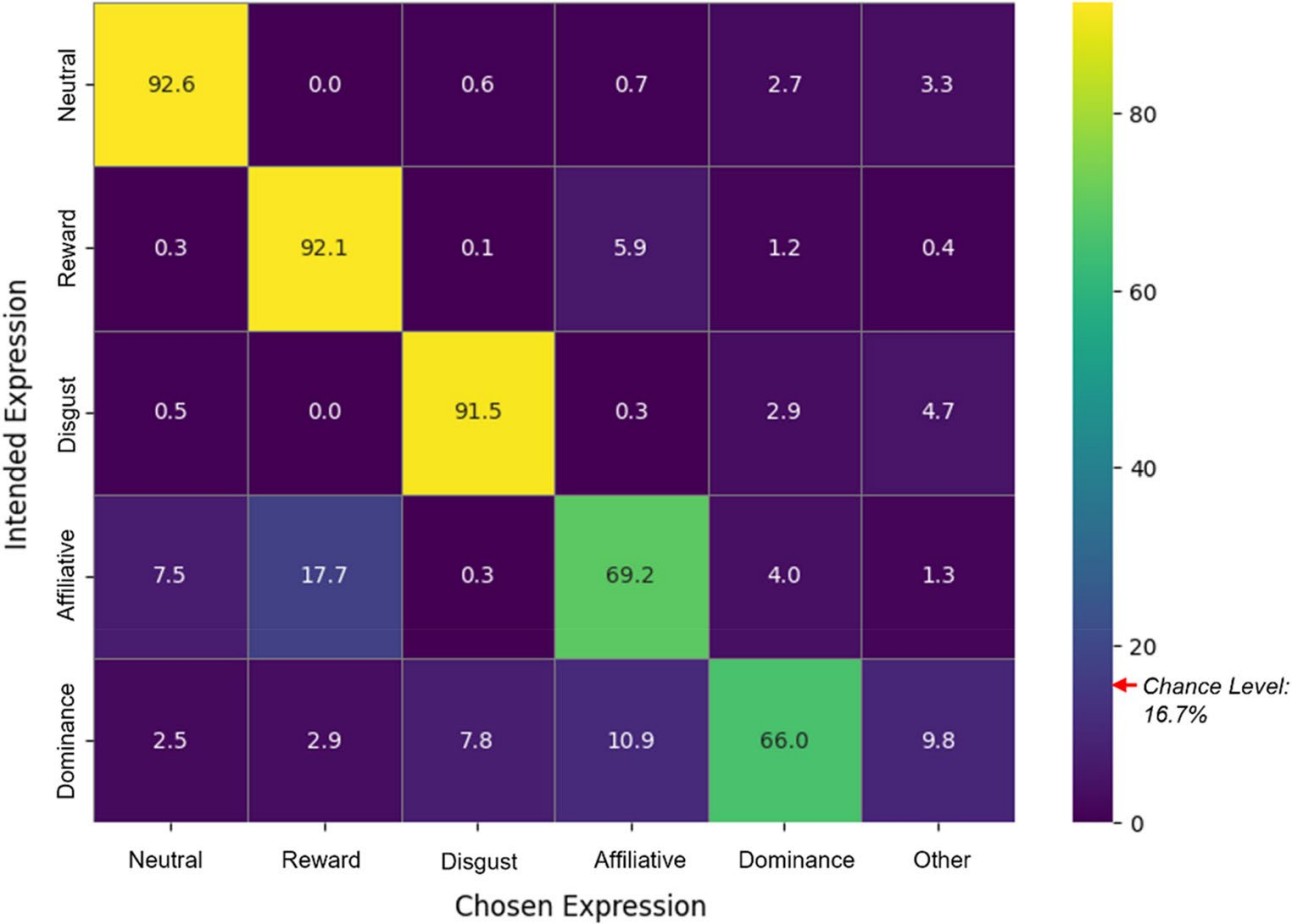
Confusion matrix: percentage of chosen emotions per intended emotional expression. *Note.* The diagonal elements represent correct classifications, while the off-diagonal elements show the distribution of misclassifications. The colour intensity corresponds to the percentage values, with brighter colours indicating higher percentages. Chance level is 16.7%, marked by a red arrow.

According to Bänziger et al. (2012) a Confusion Matrix is an appropriate and complete way to provide all the relevant information about potential bias since different attempts to propose indices that are corrected for chance guessing and/or response bias (e.g., the UHR suggested by Wagner, 1993) generally mask the origin of the potential biases and do not allow to take these into account in interpreting the results. Thus, we also present the confusion matrix (see Fig. 3) representing the classification results of emotional facial expressions, illustrating the percentage of chosen emotional categories (e.g., reward, disgust, affiliative, dominance, neutral, other) for each intended emotional expression.

A close look at the confusion matrix reveals several notable patterns in the participants’ responses to different facial expressions. Faces with intended affiliative smiles were frequently confused with neutral expressions (7.5%) and reward smiles (17.7%), suggesting significant perceptual overlap between these expressions. Similarly, intended dominance expressions were misclassified as affiliative (10.9%) disgust (7.8%) or others (9.8%), highlighting some confusion in identifying dominance-related expressions. Reward expressions were correctly identified in most cases, though there was some misclassification as affiliative (5.9%).

### Arousal and plausibility

In our study, the mean scores of the arousal ratings were 2.63 (*SD =* 1.66) for neutral expressions, 4.50 (*SD =* 1.60) for affiliative, 5.44 (*SD =* 1.66) for disgust, 5.29 (*SD =* 1.59) for dominance, and 6.72 (*SD =* 1.39) for reward. Mean scores for plausibility ratings were 5.21 (*SD =* 1.26) for neutral expressions, 4.66 (*SD =* 1.39) for affiliative, 4.23 (*SD =* 1.41) for disgust, 4.06 (*SD =* 1.43) for dominance, and 5.02 (*SD =* 1.33) for reward.

To compare the arousal and plausibility scores across expression categories, linear mixed-effects models (LMM) were fitted to these scores with fixed effects of expression category, group, rater gender, model gender, and image version, and a random intercept for CASE. Models were fitted using restricted maximum likelihood (REML). The intercept was estimated at 2.759 (*SE =* 0.223, *t*(≈ 123.9) = 12.36, *p <* 0.001), representing the predicted arousal score for the reference level (neutral) of expression category. Compared with neutral expressions, the fixed effects estimates for expression category were as follows: affiliative: 1.865 (*SE =* 0.0408, *t* = 45.73, *p <* 0.001); disgust: 2.808 (*SE =* 0.0408, *t* = 68.88, *p <* 0.001); dominance: 2.661 (*SE =* 0.0408, *t* = 65.26, *p <* 0.001); and reward: 4.088 (*SE =* 0.0408, *t* = 100.26, *p <* 0.001). Tukey‐adjusted pairwise comparisons confirmed that ratings in the neutral condition were significantly lower than each other expression (all *p*-values *<* 0.001). In comparisons between non-neutral categories, all differences were significant (*p <* 0.001), with the exception of the disgust–dominance contrast, which, though smaller, remained statistically significant (*p =* 0.0028). Additionally, the effect of model gender was significant: images showing male faces received lower arousal ratings than those showing female faces (coefficient = − 0.145, *SE =* 0.0365, *p <* 0.001);

A similar LMM was applied to the plausibility scores. The intercept was estimated at 4.833 (*SE =* 0.190, *t*(≈ 124) = 25.46, *p <* 0.001). Compared with neutral expressions, the fixed effects estimates were − 0.553 (*SE =* 0.035, *t* = − 15.79, *p <* 0.001) for affiliative, − 0.986 (*SE =* 0.035, *t* = − 28.15, *p <* 0.001) for disgust, − 1.150 (*SE =* 0.035, *t* = − 32.84, *p <* 0.001) for dominance, and − 0.197 (*SE =* 0.035, *t* = − 5.61, *p <* 0.001) for reward. Tukey-adjusted comparisons indicated that all pairwise comparisons between expression categories were statistically significant (*p <* 0.001). Moreover, the effect of model gender was significant, with images of male faces receiving higher plausibility ratings than female faces (coefficient = 0.248, *SE =* 0.0313, *p <* 0.001). Additionally, stimulus version (original vs. mirrored) significantly affected plausibility ratings, with R-versions (mirrored images) rated slightly but significantly less plausible than L-versions (original orientation) (coefficient = − 0.169, *SE* = 0.044, *p* < 0.001).

Overall, although the Shapiro–Wilk and Levene tests for the residuals were statistically significant—likely due to the large sample sizes—visual inspection of residual plots suggested that deviations from normality and homogeneity of variance were minor. Overall, both LMM models revealed statistically significant differences in ratings across expression category levels, and the potential effects of model gender in shaping perceptual evaluations of the images.

### Interrater reliability

To assess the reliability of the ratings across participants for arousal, plausibility, and emotion categorization, we calculated Fleiss’ Kappa (for categorical data) and Intraclass Correlation Coefficients (ICCs) (for continuous data). Fleiss’ Kappa estimates the agreement among multiple raters for nominal categories (Fleiss, 1971), while ICCs provide a measure of consistency in ratings across raters (Koo & Li, 2016; Shrout & Fleiss, 1979).A linear mixed-effects model with Group (i.e., each of the 10 rater lists) as a fixed effect was first used to examine potential between-group differences. Since no significant group effects were found for any of the rating types, we report mean Kappa and ICC values collapsed across all 10 groups.

#### Fleiss’ Kappa 

The agreement between participants in categorizing emotional expressions was substantial, with an overall Fleiss’ Kappa of 0.65, indicating reliable identification of the intended expression categories.

#### ICC

To objectively select the appropriate ICCs, the model fit (deviance information criterion) was calculated for all possible ICC models. The model with the best fit to our data was ICC 2, applying a two-way cross-classified multilevel model. The reliability of the judges’ ratings for arousal and plausibility was assessed using ICC (2,1) and ICC (2, *k*). ICC (2, 1) intraclass correlation coefficient, each image is measured by each rater with reliability estimated from a single measurement; ICC (2, *k*) as before, but here reliability is calculated by imputing the average of the k raters’ measurements (Bijsterbosch et al., 2021).

For arousal, the mean ICC (2,1) was 0.44, indicating moderate agreement between individual raters, while the ICC(2,k) was 0.97, reflecting excellent reliability at the group level. For plausibility, ICC (2,1) was lower at 0.14, indicating poor reliability across individuals, but the ICC(2,k) reached 0.88, suggesting good average agreement across raters (based on standard guidelines from Koo & Li, 2016).

### Ranking of expressors

**Fig. 4:**
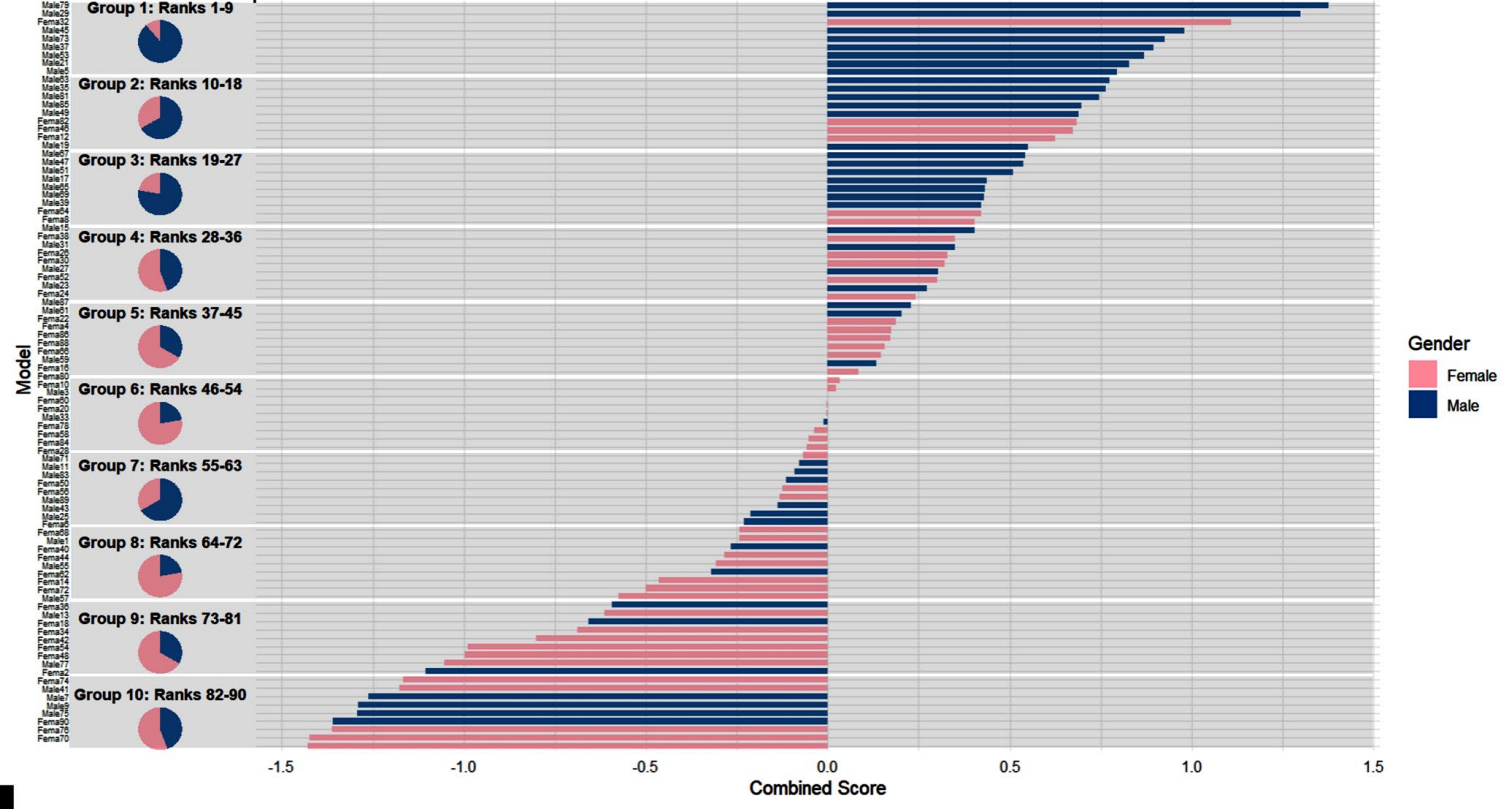
Expressor ranking for combined uhr and plausibility scores with group-wise gender distribution. *Note.* Combined scores were derived by averaging the standardized arcsine-transformed unbiased hit rates (UHR) (across all expressions) and plausibility ratings. The figure ranks all 90 expressors, divided into ten subgroups of nine expressors each, based on their combined scores. Shaded background sections on the bar chart indicate the rank ranges for each subgroup. The horizontal bar chart shows the combined scores (x-axis) and model names (y-axis), while the pie charts illustrate the gender distribution within each group, with blue representing male expressors and pink representing female expressors

In order to assess the relative quality of the expressions derived for the different individuals on which the expressions were based we ranked the performance of all expressors. At first we calculated the UHR and plausibility scores across all emotion categories per expressor. As described above, the UHR values had been arcsine-transformed to stabilize variance. A composite score was then derived by averaging the Z-scores of the arcsine-transformed UHRs and the plausibility scores. Our 90 expressors were ranked based on this composite score, grouped into ten rank-based categories, with gender distributions illustrated through pie charts (see Fig. 4). See Appendix for an overview of all individual expressors.

## Discussion

Extending prior applications of FACSGen, the present study introduced a rigorous yet easy-to-use pipeline for synthesizing socially nuanced emotional expressions from neutral portraits of real individuals. Using variants of smiles as examples, we validated the stimuli generated through this pipeline via recognition and subjective ratings. (With permission from us and from the owners of the neutral face databases used here, researchers can directly employ the validated stimuli or apply the current pipeline to create other fine-grained emotional expressions of interest.)

Overall, recognition accuracy of the stimuli was high—especially for reward and basic expressions—whereas affiliative and dominance smiles were more frequently confused with related categories, reflecting their subtle overlapping nature. Arousal and plausibility ratings also varied systematically by expression type and expressor gender.

Together, this study provides both a validated methodological framework and a reliable set of socially nuanced smile stimuli, thereby enriching research tools for studying emotional communication and social perception.

### Emotion recognition accuracy

Recognition accuracy is a crucial measure of validity for emotional stimulus sets. In our validation study, we systematically evaluated how well emotional expressions synthesized from neutral images of real expressors—particularly subtle variants of smiles—can be identified. To this aim, we employed Simple Hit Rates (SHR) and Unbiased Hit Rates (UHR) to evaluate overall recognition accuracy, and visualized response tendencies using a confusion matrix illustrating perceptual overlaps among certain expressions.

The mean SHR for neutral expressions was remarkably high (92.6%), surpassing previously reported hit rates, such as the 87% reported by Ebner et al. (2010). This likely reflects the fact that many of the neutral-expression portraits used here were sourced from validated datasets and had already undergone a selection process based on recognizability. The demographic profile of our participants—exclusively younger adults—may have also contributed, as younger individuals typically perform better in facial emotion recognition than older adults.

Among emotional expressions, disgust demonstrated high recognition accuracy (91.5%), exceeding previously reported benchmarks such as the 68.6% accuracy reported by Krumhuber et al. (2012) during the initial validation of FACSGen 2.0-generated disgust expressions. The ‘happiness’ category used in previous studies closely aligns with the reward smiles in our study. The mean recognition accuracy for reward smiles in the present study (92.1%) was somewhat higher than the happiness recognition accuracy reported by Krumhuber et al. (2012) (88.46%). These findings highlight the efficacy of using carefully controlled AU configurations in FACSGen 2.0 to generate highly distinguishable emotional stimuli.

**Fig. 5:**
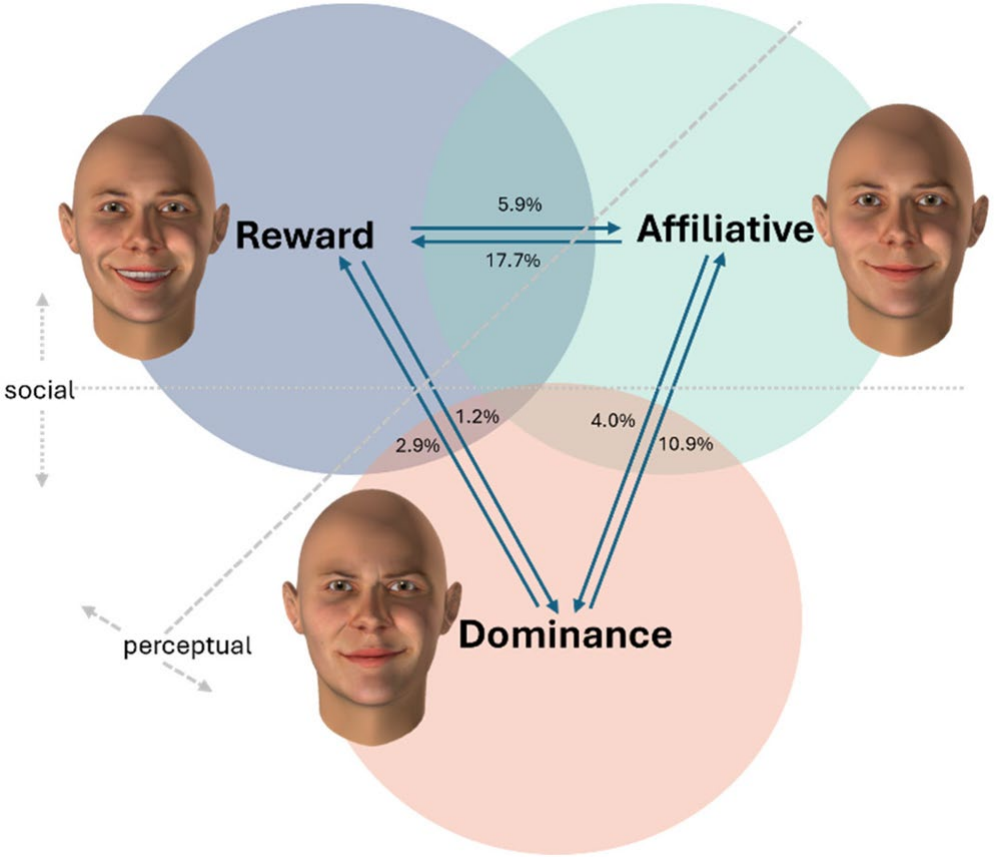
Visualization of overlap among smile types. *Note*: The diagram illustrates the relative confusion rates between reward, affiliative, and dominance smiles based on recognition data. Arrows indicate the direction of misclassification, with the corresponding percentages shown along each arrow. Circle overlap conceptually represents shared perceptual and social features. Reward–dominance smiles show minimal overlap (≈ 2%), reward–affiliative smiles the highest (≈ 11.8%), and affiliative–dominance smiles an intermediate level (≈ 7.5%)

The confusion matrix reveals overlaps among subtle smiles, aligning with Rychlowska et al. (2017), who noted that subtle smile variations present both perceptual and cognitive challenges. Although perceptual and social factors are inherently intertwined in the categorization of facial expressions (Ong et al., 2015), the present data allow for some tentative inferences about their relative contributions (see Fig. 5 for a visual framework that illustrates the overlap patterns among different types of smiles). Overall, Our results indicate that perceptual and social factors jointly influence smile categorization accuracy. When two smiles differ in both perceptual features and social meanings, they are most easily distinguished; when similar in both aspects, they are most confusable; and when perceptual features overlap but social functions differ (as with affiliative versus dominance smiles), social signal differences help compensate for perceptual similarity-induced confusion.

Specifically, the clearest distinction emerged between reward and dominance smiles, which showed only about 2% classification errors (averaged across directions). These two expressions differ markedly both perceptually and socially. As shown in Table 1, reward smiles, besides activation of the Lip Corner Puller (AU12), include strong activation of the Cheek Puffer (AU13)—typically accompanied by the Dimpler (AU14) and visible teeth. In contrast, dominance smiles lack activation of the Cheek Puffer (AU13) and visible teeth; even when the Dimpler (AU14) is activated, it tends to appear unilaterally, producing a characteristic asymmetrical appearance. Socially, these expressions signal positive internal affect versus assertive social superiority, respectively; At the other end of the spectrum, the lowest separability was observed between reward and affiliative smiles, with a mean classification error of approximately 11.8%. These expressions share perceptual similarity—with activation of the Lip Corner Puller (AU12), Cheek Puffer (AU13), and symmetrical Dimpler (AU14) contributing to perceptual ambiguity—and similar positive social meanings, which together increase the likelihood of confusion. Hence, their confusion appears to stem from both overlapping perceptual features and convergent social signalling; Between these extremes, affiliative and dominance smiles showed an intermediate confusion level (about 7.5%). While both share activation of the Lip Corner Puller (AU12) and Dimpler (AU14), they differ in their social functions: affiliative smiles express friendly intent and bonding, whereas dominance smiles convey status assertion or social control. This social contrast likely facilitates partial differentiation despite perceptual overlap.

The observations from SHR and the confusion matrix were supported by the Unbiased Hit Rate (UHR) analyses, which account for both presentation and response biases. Neutral, disgust, and reward expressions consistently yielded higher recognition performance compared to affiliative and dominance smiles, though importantly, accuracy remained far above chance levels for all emotion categories employed here.

In summary, our findings confirm the effectiveness of predefined AU configurations in producing distinguishable emotional expressions via FACSGen 2.0, while revealing the inherent perceptual and social-cognitive challenges in recognizing subtle smile variants.

### Arousal and plausibility

Our analyses of arousal ratings revealed clear distinctions among expression categories that align with expectations. Consistent with previous research (e.g., Goeleven et al., 2008), neutral expressions elicited the lowest arousal ratings for the image (*M =* 2.63), serving as an effective baseline for other emotional expressions. In contrast, disgust expressions produced substantially higher arousal ratings (*M =* 5.44), reflecting their established role in signalling negative affective states (Rozin et al., 1994).

Notably, our primary interest concerned the subtle variants of smiles. Reward smiles received the highest arousal ratings (*M =* 6.72), which is consistent with their communicative function of conveying positive emotional intensity and social engagement (Sauter, 2010). Affiliative (*M =* 4.50) and dominance smiles (*M =* 5.29) elicited intermediate arousal levels, consistent with their more nuanced communicative roles. Affiliative smiles, aiming primarily at signalling warmth and cooperation, understandably evoke more moderate arousal. Dominance smiles, signalling social status or assertiveness, produced higher arousal than affiliative but lower than reward smiles, reflecting their social signalling function as documented in prior studies (Rychlowska et al., 2017).

Plausibility ratings about how likely it was that the synthetic images were derived from pictures of real people were conducted to assess the apparent naturalness of the stimuli, which is very important for their usefulness in experimental studies (Diel et al., 2021). The plausibility ratings indicated distinctions among the expressions. Neutral expressions were perceived as most plausibly human-based (*M =* 5.21), given their minimal modifications and frequent occurrence in natural interactions. Synthetic reward smiles (*M =* 5.02) obtained high plausibility ratings comparable to neutral faces, consistent with findings that smiles reflecting genuine positive affect tend to be perceived as natural and authentic (Ekman & Friesen, 1982). In contrast, disgust (*M =* 4.23) and dominance smiles (*M =* 4.06) received lower plausibility scores, possibly due to their reliance on less frequently encountered Action Units (e.g., AU9 and AU10), making them less common in everyday facial displays and thus perceived as less genuine or natural (Dyck et al., 2008). The plausibility of affiliative smiles (*M =* 4.66) fell in-between, suggesting that subtler smile expressions, while socially meaningful, might challenge observers’ perceptions of naturalness due to their ambiguity or less frequent occurrence (Rychlowska et al., 2017).

Beyond expression type, we also observed systematic differences based on the model’s gender, despite identical AU parameters across expressors. Specifically, female expressors elicited significantly higher arousal ratings compared to male expressors, aligning with previous research suggesting observers perceive female emotional expressions as more affectively intense or expressive (Hess et al., 2000; Plant et al., 2000). Conversely, male expressors received higher plausibility ratings, indicating their emotional expressions were perceived as more genuine or believable, though relatively unnatural hairstyles assigned to female expressors might partially explain this finding. Future studies could address this by concealing external facial features with oval masks or using more realistic hairstyles. Alternatively, societal stereotypes or expectations may also contribute to this result, as emotional displays by men could be seen as more intentional or credible, whereas heightened expressivity in women might sometimes be interpreted as exaggerated or performative (Kring & Gordon, 1998).

Overall, these findings confirm that our synthesis method successfully generates expressions that vary meaningfully along both arousal and plausibility dimensions. The clear differentiation between reward, affiliative, and dominance smiles highlights the perceptual relevance of subtle AU variations in conveying affective meaning. Moreover, the observed gender differences in perceived intensity and naturalness, despite identical AU settings, suggest that visual appearance and social expectations interact in shaping emotion perception. Together, these results underscore the importance of considering both low-level visual cues and higher-order social factors in the design and validation of emotional facial stimuli.

### Interrater reliability

Our analysis of interrater reliability indicates a generally robust consistency among participants. The overall Fleiss’ Kappa value of 0.65 for emotion categorization demonstrates substantial agreement (Landis & Koch, 1977), suggesting that participants were consistent in identifying the intended expressions provided in the current study. This level of agreement supports the validity of our categorization process; for arousal ratings, the single-rater reliability, as measured by ICC (2,1), was moderate at 0.44, while the average reliability across participants, ICC (2, *k*), reached an excellent level of 0.97. This disparity highlights that, although individual ratings might vary, the aggregate mean of multiple participants yields highly reliable arousal estimates; In the case of plausibility ratings, a similar pattern emerged: the single-rater ICC (2,1) was low (0.14), indicating considerable variability at the individual level. However, the aggregate reliability (ICC (2, *k*) was strong at 0.88. These results underscore the importance of aggregating ratings in studies of this kind, as group averages effectively mitigate individual differences and enhance overall reliability.

Collectively, these reliability indices confirm that our methodological approach yields consistent ratings across multiple evaluators, thereby reinforcing the robustness of our experimental design and the validity of our subsequent analyses.

### Ranking of expressors

Additionally, to further evaluate and compare the overall quality of synthesized stimuli, we developed a composite ranking score for each expressor by integrating standardized unbiased hit rates (UHR) and plausibility ratings. This composite measure enabled us to systematically identify which expressors performed optimally across multiple evaluation criteria, providing practical guidance for researchers selecting the most effective emotional stimuli for experimental purposes. The ranking also highlighted potential differences related to model gender distribution with images derived from male expressors scoring higher than female expressors, which could inform future studies aimed at balancing stimuli to mitigate biases or investigate gender-specific effects in emotion perception. We speculate that the generally smoother appearance of female faces makes it harder to effectively apply the AU manipulation by the software employed.

### Perspectives and limitations

The current procedure offers researchers considerable flexibility, allowing image selection based on various parameters—such as model gender, hemiface, expression category, recognition accuracy, arousal, and plausibility—to meet diverse research needs. Naturally, the pipeline can be extended to generate other or compound emotions, provided that the intended emotional expressions can be precisely defined in terms of Action Units (AUs). Moreover, our open-source code^2^ base enables researchers to independently validate their own stimuli.

Another promising avenue for future research is the inclusion of dynamic facial expressions, which can be readily created using FACSGen 2.0. Prior research has shown that dynamic expressions enhance affect recognition, increase perceived authenticity, and aid in distinguishing genuine from posed expression (Krumhuber et al., 2013). Additionally, expanding the database to include faces from more diverse racial backgrounds is both feasible and beneficial. Because our method operates on neutral base portraits, which are readily available or easily obtained, extending this framework requires no specialized acting skills or training on the part of expressors.

Several limitations of the present work should be acknowledged. First, because our method is based on neutral portraits sourced from different existing databases, any stimuli originating from external databases are available only by contacting the respective database owners due to redistribution restrictions (see Appendix). To enhance transparency and reproducibility, we have uploaded representative.fda configuration files to our OSF repository, which demonstrate the Action Unit intensity settings used for each expression category. The complete set of.fda files is available upon request. Researchers interested in using the validated stimuli should contact us for permission and access details. However, since the validation analysis code is open-source, researchers can easily apply the synthesis pipeline and validation procedures to their own image sources.

Second, although mirrored (R-version) and original (L-version) images did not differ in recognition accuracy or arousal, plausibility ratings were slightly lower for mirrored versions, especially for dominance and reward smiles. To objectively acknowledge and accurately report this observed difference, we decided to treat the two versions separately instead of merging the mirrored and original versions as initially intended. Consequently, the number of raters per expression per individual was reduced from 26 to 13. While this sample size per image aligns with typical standards in facial-expression validation studies (Delicato, 2020), it nonetheless raises potential concerns regarding the stability and generalizability of ratings at the item level. Future research involving mirrored versions or subtle perceptual differences should therefore consider recruiting a greater number of participants per image version to enhance the robustness and representativeness of individual stimulus ratings. The observed plausibility difference likely reflects subtle lighting asymmetries combined with observers’ inherent preference for the left side of the face (Lindell, 2013; Powell & Schirillo, 2009). Future research might explore under what conditions such lateralization effects significantly influence plausibility without affecting recognition accuracy or perceived arousal.

Third, our approach of uniformly synthesizing expressions from neutral faces, while offering clear advantages for experimental control, does not account for individual differences in facial musculature or expressive range of the models. As noted by Elfenbein (2017), even under highly controlled conditions, individual differences in expressive style remain perceptible to observers. Although our pipeline technically allows for individualized AU intensity adjustments, the current validation employed uniform synthesis across all faces to maintain standardization. Future researchers interested in person-specific expressive differences may extend this framework accordingly.

## Conclusions

This study presents a cost-effective pipeline for generating high-quality emotional stimuli from neutral facial images while preserving individual identity. The synthesized expressions—reward, affiliative, and dominance smiles, disgust, and neutral—were validated across emotional content, arousal, and plausibility. Notably, the confusions between affiliative and dominance smiles underscore the interpretive complexity of socially nuanced expressions. Together, these findings highlight the challenges of perceiving subtle affective signals and offer a validated, scalable tool to support future research on facial emotion recognition and social communication.

## Data Availability

The study was preregistered on OSF. The preregistration and all anonymized rating data are available at https://osf.io/j4dfr/?view_only=8f6a7b25d26f4b79a6764eb398594285(Open-Ended Registration, registered on August 7, 2024). The full set of in-house-derived stimuli is available upon request from the corresponding author. Any materials originating from external databases remain available via their original owners due to redistribution restrictions.

## Appendix

**Table 4:** Performance metrics for female expressors

Expressor	Expression category	UHR	Simple hit rate	Arousal score	Plausibility score	Source	Match code
Fema2	affiliative	0.53	0.65	4.27	4.12	FACES	182_y_f_n_a
Fema2	disgust	0.83	0.96	6.42	3.23	FACES	182_y_f_n_a
Fema2	dominance	0.48	0.58	5.35	4.04	FACES	182_y_f_n_a
Fema2	reward	0.68	0.88	6.69	4.62	FACES	182_y_f_n_a
Fema2	neutral	0.86	0.96	2.85	5.23	FACES	182_y_f_n_a
Fema4	affiliative	0.58	0.73	4.46	4.38	in-house	f71_neu_1
Fema4	disgust	1	1	5.35	3.58	in-house	f71_neu_1
Fema4	dominance	0.69	0.69	5.54	4.12	in-house	f71_neu_1
Fema4	reward	0.75	0.96	6.62	4.69	in-house	f71_neu_1
Fema4	neutral	0.93	1	2.88	5.27	in-house	f71_neu_1
Fema6	affiliative	0.55	0.62	4.04	4.31	FACES	173_y_f_n_a
Fema6	disgust	0.93	1	6.08	4	FACES	173_y_f_n_a
Fema6	dominance	0.66	0.73	5	4.19	FACES	173_y_f_n_a
Fema6	reward	0.79	0.92	6.77	4.62	FACES	173_y_f_n_a
Fema6	neutral	0.76	1	2.69	5.38	FACES	173_y_f_n_a
Fema8	affiliative	0.66	0.73	3.92	4.58	FACES	171_y_f_n_a
Fema8	disgust	0.74	0.85	5.27	4.08	FACES	171_y_f_n_a
Fema8	dominance	0.58	0.62	4.42	4.19	FACES	171_y_f_n_a
Fema8	reward	0.86	0.96	6.73	5.08	FACES	171_y_f_n_a
Fema8	neutral	0.69	0.85	3.46	5.38	FACES	171_y_f_n_a
Fema10	affiliative	0.74	0.85	4.04	4.38	MR2	wf01
Fema10	disgust	0.89	0.96	5.81	3.96	MR2	wf01
Fema10	dominance	0.59	0.69	5	3.88	MR2	wf01
Fema10	reward	0.79	0.92	6.65	4.85	MR2	wf01
Fema10	neutral	0.86	0.96	2.69	5.15	MR2	wf01
Fema12	affiliative	0.56	0.65	4.08	5.04	FACES	152_y_f_n_a
Fema12	disgust	0.74	0.85	5.46	4.58	FACES	152_y_f_n_a
Fema12	dominance	0.73	0.73	4.92	4.54	FACES	152_y_f_n_a
Fema12	reward	0.7	0.88	6.54	5	FACES	152_y_f_n_a
Fema12	neutral	0.76	0.92	3.12	5.54	FACES	152_y_f_n_a
Fema14	affiliative	0.69	0.73	4.31	4.08	London	014_03
Fema14	disgust	0.81	0.81	6.46	3.69	London	014_03
Fema14	dominance	0.57	0.77	5.62	3.65	London	014_03
Fema14	reward	0.8	0.96	6.69	4.69	London	014_03
Fema14	neutral	0.86	0.96	2.96	5.04	London	014_03
Fema16	affiliative	0.48	0.69	4.15	4.77	CFD	CFD-WF-011-002-N
Fema16	disgust	0.85	0.88	5.31	4.69	CFD	CFD-WF-011-002-N
Fema16	dominance	0.55	0.62	4.96	4.46	CFD	CFD-WF-011-002-N
Fema16	reward	0.76	0.92	7	4.96	CFD	CFD-WF-011-002-N
Fema16	neutral	0.84	1	3.19	5.5	CFD	CFD-WF-011-002-N
Fema18	affiliative	0.52	0.62	4.12	3.88	FACES	134_y_f_n_a
Fema18	disgust	0.89	0.96	5.5	3.73	FACES	134_y_f_n_a
Fema18	dominance	0.55	0.62	4.73	3.81	FACES	134_y_f_n_a
Fema18	reward	0.76	0.92	6.69	5.04	FACES	134_y_f_n_a
Fema18	neutral	0.78	0.96	2.88	5.15	FACES	134_y_f_n_a
Fema20	affiliative	0.63	0.73	4.58	4.62	in-house	f15_neu_1
Fema20	disgust	0.86	0.96	5.5	3.88	in-house	f15_neu_1
Fema20	dominance	0.65	0.65	4.77	3.88	in-house	f15_neu_1
Fema20	reward	0.69	0.92	6.65	4.96	in-house	f15_neu_1
Fema20	neutral	0.85	0.92	2.81	5.12	in-house	f15_neu_1
Fema22	affiliative	0.77	0.77	4.58	4.04	FACES	115_y_f_n_a
Fema22	disgust	0.81	1	5.62	4.31	FACES	115_y_f_n_a
Fema22	dominance	0.54	0.54	5.85	3.35	FACES	115_y_f_n_a
Fema22	reward	0.84	1	7.04	4.77	FACES	115_y_f_n_a
Fema22	neutral	0.89	0.92	3.19	5.23	FACES	115_y_f_n_a
Fema24	affiliative	0.62	0.77	4.12	4.31	London	019_03
Fema24	disgust	0.74	0.85	5.5	4.46	London	019_03
Fema24	dominance	0.62	0.65	4.85	3.73	London	019_03
Fema24	reward	0.76	0.92	6.62	5.54	London	019_03
Fema24	neutral	0.9	1	1.96	5.58	London	019_03
Fema26	affiliative	0.66	0.69	4.58	4.69	CFD	CFD-WF-033-002-N
Fema26	disgust	0.7	0.77	6.27	4.69	CFD	CFD-WF-033-002-N
Fema26	dominance	0.52	0.69	5.5	3.69	CFD	CFD-WF-033-002-N
Fema26	reward	0.79	0.92	6.88	4.96	CFD	CFD-WF-033-002-N
Fema26	neutral	0.82	0.92	3.23	5.73	CFD	CFD-WF-033-002-N
Fema28	affiliative	0.68	0.81	4.65	4.65	London	027_03
Fema28	disgust	0.8	0.96	5.31	4.04	London	027_03
Fema28	dominance	0.62	0.65	5.65	2.96	London	027_03
Fema28	reward	0.73	0.88	6.35	4.85	London	027_03
Fema28	neutral	0.96	0.96	2.54	5.5	London	027_03
Fema30	affiliative	0.73	0.73	4.31	5.31	FACES	090_y_f_n_a
Fema30	disgust	0.76	1	5.31	4.23	FACES	090_y_f_n_a
Fema30	dominance	0.54	0.58	4.73	3.54	FACES	090_y_f_n_a
Fema30	reward	0.85	0.92	6.19	5.58	FACES	090_y_f_n_a
Fema30	neutral	0.78	0.96	1.73	5.15	FACES	090_y_f_n_a
Fema32	affiliative	0.67	0.77	5.04	4.35	in-house	f25_neu_1
Fema32	disgust	0.81	0.85	5.38	4.27	in-house	f25_neu_1
Fema32	dominance	0.81	0.85	5.23	4.23	in-house	f25_neu_1
Fema32	reward	0.78	0.96	6.54	5.19	in-house	f25_neu_1
Fema32	neutral	0.96	0.96	2.35	5.35	in-house	f25_neu_1
Fema34	affiliative	0.69	0.73	4.58	4.19	FACES	071_y_f_n_a
Fema34	disgust	0.67	0.92	5.62	3.85	FACES	071_y_f_n_a
Fema34	dominance	0.58	0.58	5.15	2.88	FACES	071_y_f_n_a
Fema34	reward	0.82	0.92	6.08	4.54	FACES	071_y_f_n_a
Fema34	neutral	0.9	1	2.62	4.92	FACES	071_y_f_n_a
Fema36	affiliative	0.62	0.62	4.04	4.42	FACES	069_y_f_n_a
Fema36	disgust	0.73	0.96	5.69	4.23	FACES	069_y_f_n_a
Fema36	dominance	0.38	0.38	5.42	3.27	FACES	069_y_f_n_a
Fema36	reward	0.9	1	6.96	4.69	FACES	069_y_f_n_a
Fema36	neutral	0.78	0.96	2.46	5.19	FACES	069_y_f_n_a
Fema38	affiliative	0.59	0.69	4.42	5.19	in-house	f72_neu_1
Fema38	disgust	0.92	0.96	4.54	3.96	in-house	f72_neu_1
Fema38	dominance	0.66	0.69	5.31	3.46	in-house	f72_neu_1
Fema38	reward	0.7	0.88	6.54	5.08	in-house	f72_neu_1
Fema38	neutral	0.79	0.92	2.38	5.5	in-house	f72_neu_1
Fema40	affiliative	0.48	0.65	4.27	4.69	FACES	054_y_f_n_a
Fema40	disgust	0.89	0.96	5.12	3.73	FACES	054_y_f_n_a
Fema40	dominance	0.81	0.85	5.96	3.65	FACES	054_y_f_n_a
Fema40	reward	0.58	0.81	6.46	4.65	FACES	054_y_f_n_a
Fema40	neutral	0.96	0.96	2.12	5.04	FACES	054_y_f_n_a
Fema42	affiliative	0.47	0.62	4.65	4.5	FACES	048_y_f_n_a
Fema42	disgust	0.85	0.88	5.69	3.88	FACES	048_y_f_n_a
Fema42	dominance	0.52	0.62	6.31	3.12	FACES	048_y_f_n_a
Fema42	reward	0.61	0.81	6.62	3.85	FACES	048_y_f_n_a
Fema42	neutral	0.73	0.77	2.92	5.35	FACES	048_y_f_n_a
Fema44	affiliative	0.61	0.81	4.88	5.15	London	039_03
Fema44	disgust	0.81	0.85	4.88	4	London	039_03
Fema44	dominance	0.62	0.65	5.27	3.62	London	039_03
Fema44	reward	0.63	0.81	6.69	4.54	London	039_03
Fema44	neutral	0.72	0.85	2.35	5.54	London	039_03
Fema46	affiliative	0.54	0.69	4.92	5.31	CFD	CFD-WF-005–010-N
Fema46	disgust	0.89	0.92	5.08	3.85	CFD	CFD-WF-005–010-N
Fema46	dominance	0.73	0.77	5.65	4.12	CFD	CFD-WF-005–010-N
Fema46	reward	0.66	0.88	7.19	5.19	CFD	CFD-WF-005–010-N
Fema46	neutral	0.85	0.88	2.65	5.77	CFD	CFD-WF-005–010-N
Fema48	affiliative	0.48	0.65	4.31	4.62	FACES	028_y_f_n_a
Fema48	disgust	0.86	0.96	5.62	4.12	FACES	028_y_f_n_a
Fema48	dominance	0.56	0.65	5.88	3.27	FACES	028_y_f_n_a
Fema48	reward	0.67	0.77	6.5	3.92	FACES	028_y_f_n_a
Fema48	neutral	0.78	0.88	2.5	4.85	FACES	028_y_f_n_a
Fema50	affiliative	0.58	0.73	4.54	4.62	London	090_03
Fema50	disgust	0.93	1	4.88	4.38	London	090_03
Fema50	dominance	0.47	0.54	4.65	3.81	London	090_03
Fema50	reward	0.72	0.85	6.88	4.69	London	090_03
Fema50	neutral	0.83	0.96	2.15	5.46	London	090_03
Fema52	affiliative	0.56	0.65	4.92	4.96	FACES	020_y_f_n_a
Fema52	disgust	0.82	0.92	4.88	4.35	FACES	020_y_f_n_a
Fema52	dominance	0.66	0.73	5.62	3.42	FACES	020_y_f_n_a
Fema52	reward	0.78	0.88	6.77	4.88	FACES	020_y_f_n_a
Fema52	neutral	0.82	0.92	2.5	5.27	FACES	020_y_f_n_a
Fema54	affiliative	0.5	0.69	4.54	4.23	FACES	010_y_f_n_a
Fema54	disgust	0.92	0.96	5.04	4.12	FACES	010_y_f_n_a
Fema54	dominance	0.4	0.46	5	3.08	FACES	010_y_f_n_a
Fema54	reward	0.85	0.92	6.27	4.23	FACES	010_y_f_n_a
Fema54	neutral	0.76	0.92	1.96	5	FACES	010_y_f_n_a
Fema56	affiliative	0.51	0.58	4	4.42	London	097_03
Fema56	disgust	0.79	0.92	5.58	3.88	London	097_03
Fema56	dominance	0.78	0.85	5.62	4.42	London	097_03
Fema56	reward	0.76	0.92	6.88	4.88	London	097_03
Fema56	neutral	0.83	0.96	2.31	5	London	097_03
Fema58	affiliative	0.69	0.85	3.81	3.38	London	102_03
Fema58	disgust	0.81	0.85	5.42	4.12	London	102_03
Fema58	dominance	0.73	0.77	5.31	4.12	London	102_03
Fema58	reward	0.78	0.88	6.88	4.81	London	102_03
Fema58	neutral	0.83	0.96	2.19	5.15	London	102_03
Fema60	affiliative	0.7	0.77	4.42	4.62	in-house	f47_neu_1
Fema60	disgust	0.8	0.96	5.92	4.27	in-house	f47_neu_1
Fema60	dominance	0.53	0.65	5.77	3.88	in-house	f47_neu_1
Fema60	reward	0.79	0.92	6.96	5.15	in-house	f47_neu_1
Fema60	neutral	0.85	0.92	2.5	5.12	in-house	f47_neu_1
Fema62	affiliative	0.47	0.62	4.38	4.15	London	118_03
Fema62	disgust	0.85	0.88	5.54	4.38	London	118_03
Fema62	dominance	0.62	0.69	5.08	4.12	London	118_03
Fema62	reward	0.86	0.96	6.77	4.38	London	118_03
Fema62	neutral	0.53	0.73	2.77	5.23	London	118_03
Fema64	affiliative	0.59	0.69	4.04	4.92	CFD	CFD-WF-001–003-N
Fema64	disgust	0.77	0.81	6.04	4.5	CFD	CFD-WF-001–003-N
Fema64	dominance	0.64	0.77	6	4.42	CFD	CFD-WF-001–003-N
Fema64	reward	0.76	0.92	6.73	5.31	CFD	CFD-WF-001–003-N
Fema64	neutral	0.83	0.96	2.42	5.04	CFD	CFD-WF-001–003-N
Fema66	affiliative	0.6	0.73	4.12	4.31	CFD	CFD-WF-003-003-N
Fema66	disgust	0.89	0.96	5.69	4.35	CFD	CFD-WF-003-003-N
Fema66	dominance	0.73	0.77	5.88	4.38	CFD	CFD-WF-003-003-N
Fema66	reward	0.85	0.92	6.92	4.73	CFD	CFD-WF-003-003-N
Fema66	neutral	0.78	0.96	1.92	4.73	CFD	CFD-WF-003-003-N
Fema68	affiliative	0.41	0.5	4.12	4.73	in-house	f42_neu_1
Fema68	disgust	0.86	0.96	6.08	4.62	in-house	f42_neu_1
Fema68	dominance	0.72	0.85	5.96	4.08	in-house	f42_neu_1
Fema68	reward	0.71	0.92	6.85	5	in-house	f42_neu_1
Fema68	neutral	0.82	0.92	2.5	4.88	in-house	f42_neu_1
Fema70	affiliative	0.63	0.73	4.77	2.85	CFD	CFD-WF-200-002-N
Fema70	disgust	0.74	0.81	6.5	3.5	CFD	CFD-WF-200-002-N
Fema70	dominance	0.64	0.85	7.04	3.73	CFD	CFD-WF-200-002-N
Fema70	reward	0.85	0.88	7.5	3.65	CFD	CFD-WF-200-002-N
Fema70	neutral	0.7	0.77	2.88	4.69	CFD	CFD-WF-200-002-N
Fema72	affiliative	0.59	0.65	4.88	4.38	CFD	CFD-WF-038-021-N
Fema72	disgust	0.81	0.85	6.23	4	CFD	CFD-WF-038-021-N
Fema72	dominance	0.56	0.85	6.23	3.77	CFD	CFD-WF-038-021-N
Fema72	reward	0.78	0.88	6.88	4.5	CFD	CFD-WF-038-021-N
Fema72	neutral	0.89	0.92	2.58	4.81	CFD	CFD-WF-038-021-N
Fema74	affiliative	0.46	0.65	4.58	4.81	CFD	CFD-WF-223-133-N
Fema74	disgust	0.82	0.92	5.69	3.65	CFD	CFD-WF-223-133-N
Fema74	dominance	0.38	0.38	4.81	3.62	CFD	CFD-WF-223-133-N
Fema74	reward	0.69	0.96	7.35	4.54	CFD	CFD-WF-223-133-N
Fema74	neutral	0.89	0.96	2.88	4.92	CFD	CFD-WF-223-133-N
Fema76	affiliative	0.38	0.54	4.92	5.19	CFD	CFD-WF-203–229-N
Fema76	disgust	0.85	0.92	5.5	3.5	CFD	CFD-WF-203–229-N
Fema76	dominance	0.37	0.46	5.65	2.85	CFD	CFD-WF-203–229-N
Fema76	reward	0.72	1	7.5	4.92	CFD	CFD-WF-203–229-N
Fema76	neutral	0.85	0.88	3.42	4.5	CFD	CFD-WF-203–229-N
Fema78	affiliative	0.49	0.62	4.96	4.92	in-house	f65_neu_1
Fema78	disgust	0.74	0.85	5.69	4.31	in-house	f65_neu_1
Fema78	dominance	0.54	0.58	5.23	3.92	in-house	f65_neu_1
Fema78	reward	0.71	0.96	7.38	4.77	in-house	f65_neu_1
Fema78	neutral	1	1	2.85	5.04	in-house	f65_neu_1
Fema80	affiliative	0.54	0.58	4.58	4.88	in-house	f66_neu_1
Fema80	disgust	0.84	1	5.77	4.73	in-house	f66_neu_1
Fema80	dominance	0.44	0.54	5.96	3.65	in-house	f66_neu_1
Fema80	reward	0.79	1	7	5.27	in-house	f66_neu_1
Fema80	neutral	0.85	0.92	2.85	5.19	in-house	f66_neu_1
Fema82	affiliative	0.77	0.77	4.65	4.46	CFD	CFD-WF-220-101-N
Fema82	disgust	0.89	0.92	5.38	4.04	CFD	CFD-WF-220-101-N
Fema82	dominance	0.59	0.69	6.27	3.81	CFD	CFD-WF-220-101-N
Fema82	reward	0.84	1	6.77	4.69	CFD	CFD-WF-220-101-N
Fema82	neutral	1	1	3.23	4.62	CFD	CFD-WF-220-101-N
Fema84	affiliative	0.56	0.73	4.38	5.08	in-house	f43_neu_1
Fema84	disgust	0.62	0.65	4.85	4.12	in-house	f43_neu_1
Fema84	dominance	0.46	0.5	5.08	3.85	in-house	f43_neu_1
Fema84	reward	0.8	0.96	6.88	4.73	in-house	f43_neu_1
Fema84	neutral	0.9	1	2.65	5.12	in-house	f43_neu_1
Fema86	affiliative	0.77	0.81	4.54	4.88	in-house	f69_neu_1
Fema86	disgust	0.79	1	5.62	4.42	in-house	f69_neu_1
Fema86	dominance	0.43	0.46	5.15	3.73	in-house	f69_neu_1
Fema86	reward	0.84	1	6.62	4.81	in-house	f69_neu_1
Fema86	neutral	0.89	0.92	2.88	5.15	in-house	f69_neu_1
Fema88	affiliative	0.48	0.65	5.12	4.77	CFD	CFD-WF-248-129-N
Fema88	disgust	0.89	0.96	4.69	4.04	CFD	CFD-WF-248-129-N
Fema88	dominance	0.62	0.62	4.69	4.12	CFD	CFD-WF-248-129-N
Fema88	reward	0.76	1	6.96	4.92	CFD	CFD-WF-248-129-N
Fema88	neutral	0.96	0.96	2.46	5.04	CFD	CFD-WF-248-129-N
Fema90	affiliative	0.55	0.62	4.85	4.12	in-house	f3_neu_1
Fema90	disgust	0.83	0.96	5.46	4.12	in-house	f3_neu_1
Fema90	dominance	0.4	0.46	5.31	3.42	in-house	f3_neu_1
Fema90	reward	0.81	1	6.58	4.73	in-house	f3_neu_1
Fema90	neutral	0.75	0.96	2.69	4.46	in-house	f3_neu_1

Note. This table summarizes recognition and rating performance for female expressors across five expression categories (affiliative, disgust, dominance, reward, and neutral). The columns include: Expressor, which identifies the female expressor (e.g., Fema2); Expression category, specifying the type of expression; UHR (Unbiased Hit Rate), which adjusts for response bias; Simple hit rate, representing the raw percentage of correct responses; Arousal score and Plausibility score, both rated on 7-point Likert scales. The Source column indicates the database from which each stimulus was derived. The Match code column shows the original filename of the corresponding stimulus. *FACES* FACES database (Ebner et al., 2010), *CFD* Chicago Face database (Ma et al., 2015), *MR2* MR2 database (Strohminger et al., 2016), *London* Face Research Lab London Set (DeBruine & Jones, 2017), *in-house* stimuli created by the authors using photographs of volunteers

**Table 5:** Performance metrics for male expressors

Expressor	Expression category	UHR	Simple hit rate	Arousal score	Plausibility score	Source	Match code
Male1	affiliative	0.52	0.62	3.88	4.54	CFD	CFD-WM-254-152-N
Male1	disgust	0.89	0.92	5.42	4	CFD	CFD-WM-254-152-N
Male1	dominance	0.66	0.73	5.12	4.19	CFD	CFD-WM-254-152-N
Male1	reward	0.62	0.88	6.19	4.92	CFD	CFD-WM-254-152-N
Male1	neutral	0.8	0.96	3.23	5.69	CFD	CFD-WM-254-152-N
Male3	affiliative	0.51	0.73	4.08	5.04	FACES	013_y_f_n_a
Male3	disgust	0.81	0.85	5.42	4.04	FACES	013_y_f_n_a
Male3	dominance	0.4	0.54	5	4.65	FACES	013_y_f_n_a
Male3	reward	0.78	0.96	6.62	5.38	FACES	013_y_f_n_a
Male3	neutral	0.86	0.96	2.65	5.69	FACES	013_y_f_n_a
Male5	affiliative	0.53	0.73	5.08	4.96	CFD	CFD-WM-032-001-N
Male5	disgust	0.92	0.92	5.96	4.27	CFD	CFD-WM-032-001-N
Male5	dominance	0.74	0.81	5.73	4.96	CFD	CFD-WM-032-001-N
Male5	reward	0.73	0.88	7.12	5.04	CFD	CFD-WM-032-001-N
Male5	neutral	0.82	0.92	3.04	5.65	CFD	CFD-WM-032-001-N
Male7	affiliative	0.48	0.58	3.88	4.58	FACES	025_y_f_n_a
Male7	disgust	0.68	0.81	6.04	4.08	FACES	025_y_f_n_a
Male7	dominance	0.42	0.73	5.54	4.35	FACES	025_y_f_n_a
Male7	reward	0.75	0.88	6.73	4.96	FACES	025_y_f_n_a
Male7	neutral	0.53	0.65	3.73	5.27	FACES	025_y_f_n_a
Male9	affiliative	0.36	0.54	4.65	4.42	CFD	CFD-WM-205-007-N
Male9	disgust	0.89	0.96	5.46	4.27	CFD	CFD-WM-205-007-N
Male9	dominance	0.48	0.73	5.77	4.23	CFD	CFD-WM-205-007-N
Male9	reward	0.66	0.85	6.46	4	CFD	CFD-WM-205-007-N
Male9	neutral	0.69	0.73	3.35	5.31	CFD	CFD-WM-205-007-N
Male11	affiliative	0.64	0.77	4.08	4.23	FACES	037_y_f_n_a
Male11	disgust	0.8	0.96	5.77	4.04	FACES	037_y_f_n_a
Male11	dominance	0.55	0.62	5.04	4	FACES	037_y_f_n_a
Male11	reward	0.75	0.88	6.65	4.92	FACES	037_y_f_n_a
Male11	neutral	0.89	0.92	2.85	5.31	FACES	037_y_f_n_a
Male13	affiliative	0.52	0.62	3.81	4.31	FACES	041_y_f_n_a
Male13	disgust	0.72	0.85	5.88	4.31	FACES	041_y_f_n_a
Male13	dominance	0.5	0.69	5.38	4.19	FACES	041_y_f_n_a
Male13	reward	0.81	0.88	6.62	5	FACES	041_y_f_n_a
Male13	neutral	0.74	0.92	3.35	5.23	FACES	041_y_f_n_a
Male15	affiliative	0.66	0.69	4.15	4.23	FACES	049_y_f_n_a
Male15	disgust	0.81	0.85	5.58	4.31	FACES	049_y_f_n_a
Male15	dominance	0.65	0.92	5.08	4.77	FACES	049_y_f_n_a
Male15	reward	0.89	0.92	7	5.23	FACES	049_y_f_n_a
Male15	neutral	0.75	0.88	3.42	5.23	FACES	049_y_f_n_a
Male17	affiliative	0.66	0.73	3.77	4.54	FACES	057_y_f_n_a
Male17	disgust	0.78	0.96	5.73	4.58	FACES	057_y_f_n_a
Male17	dominance	0.51	0.58	4.88	4.19	FACES	057_y_f_n_a
Male17	reward	1	1	6.85	4.96	FACES	057_y_f_n_a
Male17	neutral	0.81	1	2.77	5.54	FACES	057_y_f_n_a
Male19	affiliative	0.58	0.73	4.35	4.19	FACES	062_y_f_n_a
Male19	disgust	0.82	0.92	5.62	4.69	FACES	062_y_f_n_a
Male19	dominance	0.54	0.54	4.08	4.27	FACES	062_y_f_n_a
Male19	reward	0.89	0.96	6.31	5.38	FACES	062_y_f_n_a
Male19	neutral	0.84	1	2.35	5.42	FACES	062_y_f_n_a
Male21	affiliative	0.78	0.85	4.5	4.85	FACES	066_y_f_n_a
Male21	disgust	0.74	0.85	5.19	4.04	FACES	066_y_f_n_a
Male21	dominance	0.74	0.81	5.04	3.96	FACES	066_y_f_n_a
Male21	reward	0.75	0.88	6.54	5.38	FACES	066_y_f_n_a
Male21	neutral	0.89	0.96	2.31	5.46	FACES	066_y_f_n_a
Male23	affiliative	0.61	0.81	4.5	4.69	CFD	CFD-WM-011-002-N
Male23	disgust	0.81	0.88	4.65	4.31	CFD	CFD-WM-011-002-N
Male23	dominance	0.5	0.5	4.38	4.58	CFD	CFD-WM-011-002-N
Male23	reward	0.64	0.88	6.58	5.85	CFD	CFD-WM-011-002-N
Male23	neutral	0.86	0.96	2.31	5.46	CFD	CFD-WM-011-002-N
Male25	affiliative	0.54	0.69	4.62	4.5	FACES	081_y_f_n_a
Male25	disgust	0.85	0.92	5.42	4.42	FACES	081_y_f_n_a
Male25	dominance	0.46	0.5	4.23	4.46	FACES	081_y_f_n_a
Male25	reward	0.71	0.92	5.88	5.12	FACES	081_y_f_n_a
Male25	neutral	0.78	0.96	2.73	5.19	FACES	081_y_f_n_a
Male27	affiliative	0.47	0.81	5.35	5.15	CFD	CFD-WM-219-008-N
Male27	disgust	0.96	0.96	5.31	4.19	CFD	CFD-WM-219-008-N
Male27	dominance	0.39	0.42	4.69	4.88	CFD	CFD-WM-219-008-N
Male27	reward	0.68	0.88	6.31	5.54	CFD	CFD-WM-219-008-N
Male27	neutral	0.92	0.92	2.73	5.19	CFD	CFD-WM-219-008-N
Male29	affiliative	0.52	0.69	4.73	5.35	London	069_03
Male29	disgust	0.96	0.96	5.27	4.58	London	069_03
Male29	dominance	0.73	0.77	4.35	4.65	London	069_03
Male29	reward	0.75	0.96	6	5.77	London	069_03
Male29	neutral	0.93	1	2.08	5.46	London	069_03
Male31	affiliative	0.57	0.69	4.31	4.58	London	029_03
Male31	disgust	0.83	0.96	5.58	4.69	London	029_03
Male31	dominance	0.47	0.62	4.88	4.88	London	029_03
Male31	reward	0.76	0.92	6.35	5.38	London	029_03
Male31	neutral	0.81	0.88	2.65	5.5	London	029_03
Male33	affiliative	0.45	0.62	4.35	4.88	FACES	179_y_f_n_a
Male33	disgust	0.76	0.92	5.54	4.5	FACES	179_y_f_n_a
Male33	dominance	0.58	0.58	4.35	4.27	FACES	179_y_f_n_a
Male33	reward	0.83	0.96	6.23	5.46	FACES	179_y_f_n_a
Male33	neutral	0.8	0.96	2.65	4.88	FACES	179_y_f_n_a
Male35	affiliative	0.65	0.81	4.58	5.04	London	041_03
Male35	disgust	0.87	1	5.38	4.88	London	041_03
Male35	dominance	0.48	0.58	5.04	4.5	London	041_03
Male35	reward	0.7	0.88	6.35	5.92	London	041_03
Male35	neutral	0.89	0.92	2.46	5.35	London	041_03
Male37	affiliative	0.73	0.88	4.62	5.23	FACES	123_y_f_n_a
Male37	disgust	0.85	0.92	4.81	4.5	FACES	123_y_f_n_a
Male37	dominance	0.74	0.81	5.42	4.12	FACES	123_y_f_n_a
Male37	reward	0.78	0.88	6.12	5.31	FACES	123_y_f_n_a
Male37	neutral	0.74	0.85	2.27	5.46	FACES	123_y_f_n_a
Male39	affiliative	0.59	0.77	4.73	4.62	FACES	127_y_f_n_a
Male39	disgust	0.83	0.96	5.54	4.65	FACES	127_y_f_n_a
Male39	dominance	0.74	0.85	5.04	4.04	FACES	127_y_f_n_a
Male39	reward	0.75	0.88	6.62	5.5	FACES	127_y_f_n_a
Male39	neutral	0.77	0.77	2.88	5.19	FACES	127_y_f_n_a
Male41	affiliative	0.29	0.54	4.23	4.73	London	117_03
Male41	disgust	0.74	0.81	3.81	4.12	London	117_03
Male41	dominance	0.26	0.42	4.62	4.15	London	117_03
Male41	reward	0.61	0.81	6.62	5.04	London	117_03
Male41	neutral	0.85	0.92	2.38	5.5	London	117_03
Male43	affiliative	0.62	0.77	4.19	4.5	FACES	144_y_f_n_a
Male43	disgust	0.89	0.96	4.46	4.19	FACES	144_y_f_n_a
Male43	dominance	0.58	0.62	4.77	3.42	FACES	144_y_f_n_a
Male43	reward	0.74	0.85	6.15	4.35	FACES	144_y_f_n_a
Male43	neutral	0.83	0.96	2	5.31	FACES	144_y_f_n_a
Male45	affiliative	0.58	0.73	4.92	4.96	CFD	CFD-WM-235-147-N
Male45	disgust	0.92	0.96	5.54	4.27	CFD	CFD-WM-235-147-N
Male45	dominance	0.64	0.77	5.88	4.46	CFD	CFD-WM-235-147-N
Male45	reward	0.76	0.92	6.54	5.46	CFD	CFD-WM-235-147-N
Male45	neutral	0.92	0.92	2.54	5.65	CFD	CFD-WM-235-147-N
Male47	affiliative	0.43	0.65	5	5.12	FACES	153_y_f_n_a
Male47	disgust	0.96	0.96	5.15	4.58	FACES	153_y_f_n_a
Male47	dominance	0.74	0.81	5.15	4.65	FACES	153_y_f_n_a
Male47	reward	0.66	0.85	6.42	5.08	FACES	153_y_f_n_a
Male47	neutral	0.89	0.96	2.12	5.35	FACES	153_y_f_n_a
Male49	affiliative	0.55	0.77	4.38	5.19	FACES	160_y_f_n_a
Male49	disgust	0.93	1	5	4.85	FACES	160_y_f_n_a
Male49	dominance	0.65	0.65	5	4.15	FACES	160_y_f_n_a
Male49	reward	0.68	0.81	6.12	5.23	FACES	160_y_f_n_a
Male49	neutral	0.83	0.96	2.23	5.62	FACES	160_y_f_n_a
Male51	affiliative	0.77	0.81	4.54	4.73	FACES	167_y_f_n_a
Male51	disgust	0.84	1	5.42	4.42	FACES	167_y_f_n_a
Male51	dominance	0.67	0.77	5.46	3.62	FACES	167_y_f_n_a
Male51	reward	0.82	0.92	6.65	4.62	FACES	167_y_f_n_a
Male51	neutral	0.81	0.85	2.42	5.46	FACES	167_y_f_n_a
Male53	affiliative	0.62	0.77	4.46	5.04	CFD	CFD-WM-252-224-N
Male53	disgust	0.9	1	4.73	4.35	CFD	CFD-WM-252-224-N
Male53	dominance	0.77	0.81	5.12	3.96	CFD	CFD-WM-252-224-N
Male53	reward	0.76	0.92	7	5.38	CFD	CFD-WM-252-224-N
Male53	neutral	0.85	0.85	2.73	5.31	CFD	CFD-WM-252-224-N
Male55	affiliative	0.46	0.58	4.73	4.62	FACES	175_y_f_n_a
Male55	disgust	0.78	0.85	6.19	4.42	FACES	175_y_f_n_a
Male55	dominance	0.57	0.88	6.23	4.46	FACES	175_y_f_n_a
Male55	reward	0.85	0.92	6.81	5.08	FACES	175_y_f_n_a
Male55	neutral	0.74	0.81	2.23	5.19	FACES	175_y_f_n_a
Male57	affiliative	0.33	0.42	4.65	4.42	MR2	wm10
Male57	disgust	0.82	0.92	6.23	4.15	MR2	wm10
Male57	dominance	0.49	0.85	5.96	4.31	MR2	wm10
Male57	reward	0.88	0.88	7	5.19	MR2	wm10
Male57	neutral	0.7	0.77	2.42	5.19	MR2	wm10
Male59	affiliative	0.53	0.65	4.77	4.88	CFD	CFD-WM-025-002-N
Male59	disgust	0.77	0.77	6	4.15	CFD	CFD-WM-025-002-N
Male59	dominance	0.59	0.77	5.38	4.54	CFD	CFD-WM-025-002-N
Male59	reward	0.75	0.96	6.96	5.04	CFD	CFD-WM-025-002-N
Male59	neutral	0.9	1	2.08	5.54	CFD	CFD-WM-025-002-N
Male61	affiliative	0.51	0.65	4.42	5.12	CFD	CFD-WM-213-076-N
Male61	disgust	0.81	0.85	5.88	4.23	CFD	CFD-WM-213-076-N
Male61	dominance	0.59	0.77	5.31	4.23	CFD	CFD-WM-213-076-N
Male61	reward	0.8	0.96	6.96	5.31	CFD	CFD-WM-213-076-N
Male61	neutral	0.85	0.92	2.23	5.15	CFD	CFD-WM-213-076-N
Male63	affiliative	0.53	0.65	4.38	4.73	CFD	CFD-WM-009-002-N
Male63	disgust	0.88	0.88	5.38	4.15	CFD	CFD-WM-009-002-N
Male63	dominance	0.63	0.73	5.62	4.69	CFD	CFD-WM-009-002-N
Male63	reward	0.75	0.96	6.92	5.69	CFD	CFD-WM-009-002-N
Male63	neutral	0.86	0.96	2.23	5.81	CFD	CFD-WM-009-002-N
Male65	affiliative	0.5	0.69	4.69	5.04	CFD	CFD-WM-013-001-N
Male65	disgust	0.81	0.85	5.35	4.38	CFD	CFD-WM-013-001-N
Male65	dominance	0.55	0.62	5.38	4.73	CFD	CFD-WM-013-001-N
Male65	reward	0.71	0.96	6.96	5.5	CFD	CFD-WM-013-001-N
Male65	neutral	0.96	1	1.96	5.27	CFD	CFD-WM-013-001-N
Male67	affiliative	0.59	0.65	4.19	4.19	CFD	CFD-WM-022-001-N
Male67	disgust	0.96	0.96	5.19	4	CFD	CFD-WM-022-001-N
Male67	dominance	0.75	0.88	5.5	4.73	CFD	CFD-WM-022-001-N
Male67	reward	0.74	0.92	6.62	5.08	CFD	CFD-WM-022-001-N
Male67	neutral	0.89	0.96	2.15	5.12	CFD	CFD-WM-022-001-N
Male69	affiliative	0.61	0.81	4.27	4.46	CFD	CFD-WM-024-015-N
Male69	disgust	0.89	0.92	5.5	4.46	CFD	CFD-WM-024-015-N
Male69	dominance	0.62	0.65	5.12	4.15	CFD	CFD-WM-024-015-N
Male69	reward	0.76	0.92	7.12	5.23	CFD	CFD-WM-024-015-N
Male69	neutral	0.93	1	2.5	5.27	CFD	CFD-WM-024-015-N
Male71	affiliative	0.45	0.62	4.58	4.31	CFD	CFD-WM-029-023-N
Male71	disgust	0.96	0.96	5.58	4.19	CFD	CFD-WM-029-023-N
Male71	dominance	0.7	0.77	5.46	4.73	CFD	CFD-WM-029-023-N
Male71	reward	0.66	0.88	6.62	5.12	CFD	CFD-WM-029-023-N
Male71	neutral	0.87	1	2.08	5.08	CFD	CFD-WM-029-023-N
Male73	affiliative	0.54	0.69	4.69	4.96	CFD	CFD-WM-031-003-N
Male73	disgust	0.96	0.96	5.27	4.54	CFD	CFD-WM-031-003-N
Male73	dominance	0.77	0.81	5.81	4.46	CFD	CFD-WM-031-003-N
Male73	reward	0.75	0.96	6.92	5.5	CFD	CFD-WM-031-003-N
Male73	neutral	0.85	0.92	2.81	5.04	CFD	CFD-WM-031-003-N
Male75	affiliative	0.31	0.62	4.81	5.42	CFD	CFD-WM-230-131-N
Male75	disgust	0.77	0.77	4.27	4.12	CFD	CFD-WM-230-131-N
Male75	dominance	0.24	0.27	4.88	4.54	CFD	CFD-WM-230-131-N
Male75	reward	0.6	0.92	6.69	5.38	CFD	CFD-WM-230-131-N
Male75	neutral	0.64	0.85	3.08	5.19	CFD	CFD-WM-230-131-N
Male77	affiliative	0.33	0.58	5.46	4.65	CFD	CFD-WM-214-026-N
Male77	disgust	0.85	0.88	4.65	4.04	CFD	CFD-WM-214-026-N
Male77	dominance	0.28	0.35	5.19	4.15	CFD	CFD-WM-214-026-N
Male77	reward	0.65	0.96	6.73	5.5	CFD	CFD-WM-214-026-N
Male77	neutral	0.9	1	2.73	5.04	CFD	CFD-WM-214-026-N
Male79	affiliative	0.59	0.69	5.23	5.15	in-house	m22_neu_1
Male79	disgust	0.89	0.92	5.31	4.81	in-house	m22_neu_1
Male79	dominance	0.65	0.65	6	4.58	in-house	m22_neu_1
Male79	reward	0.79	1	7.38	5.58	in-house	m22_neu_1
Male79	neutral	0.81	0.88	3.19	5.08	in-house	m22_neu_1
Male81	affiliative	0.63	0.73	4.85	4.96	in-house	m43_neu_1
Male81	disgust	0.92	0.96	5.54	4.54	in-house	m43_neu_1
Male81	dominance	0.63	0.73	5.23	4.27	in-house	m43_neu_1
Male81	reward	0.78	0.96	6.88	5.42	in-house	m43_neu_1
Male81	neutral	0.92	0.96	2.77	4.65	in-house	m43_neu_1
Male83	affiliative	0.54	0.69	4.65	4.54	in-house	m50_neu_1
Male83	disgust	0.85	0.88	5.27	4.38	in-house	m50_neu_1
Male83	dominance	0.72	0.85	5.65	3.77	in-house	m50_neu_1
Male83	reward	0.78	0.96	7.23	4.81	in-house	m50_neu_1
Male83	neutral	0.81	0.81	3.23	4.62	in-house	m50_neu_1
Male85	affiliative	0.47	0.62	5.04	5.65	in-house	m58_neu_1
Male85	disgust	0.85	0.92	5.08	4.62	in-house	m58_neu_1
Male85	dominance	0.54	0.54	5.12	4.35	in-house	m58_neu_1
Male85	reward	0.74	1	7.08	5.38	in-house	m58_neu_1
Male85	neutral	0.92	0.96	2.69	4.88	in-house	m58_neu_1
Male87	affiliative	0.46	0.69	4.58	4.88	in-house	m61_neu_1
Male87	disgust	0.96	0.96	5.46	4.35	in-house	m61_neu_1
Male87	dominance	0.62	0.62	5.15	3.92	in-house	m61_neu_1
Male87	reward	0.75	0.96	6.92	5.5	in-house	m61_neu_1
Male87	neutral	0.85	0.88	2.54	4.5	in-house	m61_neu_1
Male89	affiliative	0.42	0.73	4.54	5	in-house	m65_neu_1
Male89	disgust	0.92	0.92	4.54	4.42	in-house	m65_neu_1
Male89	dominance	0.17	0.23	4.92	4.65	in-house	m65_neu_1
Male89	reward	0.76	1	6.92	5.58	in-house	m65_neu_1
Male89	neutral	1	1	2.58	5.04	in-house	m65_neu_1

Note. This table summarizes recognition and rating performance for male expressors across five expression categories (affiliative, disgust, dominance, reward, and neutral). The columns include: Expressor, which identifies the male expressor (e.g., Male1); Expression category, specifying the type of expression; UHR (Unbiased Hit Rate), which adjusts for response bias; Simple hit rate, representing the raw percentage of correct responses; Arousal score and Plausibility score, both rated on 7-point Likert scales. The Source column indicates the database from which each stimulus was derived. The Match code column shows the original filename of the corresponding stimulus. *FACES* FACES database (Ebner et al., 2010 b), *CFD* Chicago Face database (Ma et al., 2015), *MR2* MR2 database (Strohminger et al., 2016), *London* Face Research Lab London Set (DeBruine & Jones, 2017), *in-house* stimuli created by the authors using photographs of volunteers

## Footnotes

1 Please see https://osf.io/xh298 for more details.

2 Please see https://github.com/JinGao1997/Facial-Expressions-Rating-Task for more details.

## Declarations

### Public significance statements

Not every smile signals happiness—some build rapport, others assert status. We offer a rigorous yet easy-to-use pipeline, built with specialized face-editing software, that turns neutral photos into controlled sets of reward, affiliative, and dominance smiles and validates them for recognition accuracy, arousal, and plausibility, giving researchers a clear recipe for studying nuanced emotion signals. The results reveal shared cues but frequent confusions among these smiles, reminding the public that even tiny facial shifts shape what we see and convey.
